# Experiences of Patients With Cancer Using Electronic Symptom Management Systems: Qualitative Systematic Review and Meta-Synthesis

**DOI:** 10.2196/59061

**Published:** 2024-10-28

**Authors:** Siying Zhu, Yan Dong, Yumei Li, Hong Wang, Xue Jiang, Mingen Guo, Tiantian Fan, Yalan Song, Ying Zhou, Yuan Han

**Affiliations:** 1 School of Nursing Guangzhou Medical University Guangzhou China; 2 Guangzhou Institute of Cancer Research, the Affiliated Cancer Hospital Guangzhou Medical University Guangzhou China

**Keywords:** electronic symptom management systems, oncology care, access to care, symptom monitoring, self-management, patient-reported outcomes, health-related outcomes, quality of life

## Abstract

**Background:**

There are numerous symptoms related to cancer and its treatments that can affect the psychosomatic health and quality of life of patients with cancer. The use of electronic symptom management systems (ESMSs) can help patients with cancer monitor and manage their symptoms effectively, improving their health-related outcomes. However, patients’ adhesion to ESMSs decreases over time, and little is known about their real experiences with them. Therefore, it is necessary to gain a deep understanding of patients’ experiences with ESMSs.

**Objective:**

The purpose of this systematic review was to synthesize qualitative studies on the experiences of patients with cancer using ESMSs.

**Methods:**

A total of 12 electronic databases, including PubMed, Web of Science, Cochrane Library, EBSCOhost, Embase, PsycINFO, ProQuest, Scopus, Wanfang database, CNKI, CBM, and VIP, were searched to collect relevant studies from the earliest available record until January 2, 2024. Qualitative and mixed methods studies published in English or Chinese were included. The PRISMA (Preferred Reporting Items for Systematic Reviews and Meta-Analyses statement checklist) and the ENTREQ (Enhancing Transparency in Reporting the Synthesis of Qualitative Research) statement were used to improve transparency in reporting the synthesis of the qualitative research. The Critical Appraisal Skills Program (CASP) checklist was used to appraise the methodological quality of the included studies, and a meta-synthesis was conducted to interpret and synthesize the findings.

**Results:**

A total of 21 studies were included in the meta-synthesis. The experiences of patients with cancer using ESMSs were summarized into three major categories: (1) perceptions and attitudes toward ESMSs; (2) the value of ESMSs; and (3) barriers, requirements, and suggestions for ESMSs. Subsequently, 10 subcategories emerged from the 3 major categories. The meta-synthesis revealed that patients with cancer had both positive and negative experiences with ESMSs. In general, patients recognized the value of ESMSs in symptom assessment and management and were willing to use them, but they still encountered barriers and wanted them to be improved.

**Conclusions:**

This systematic review provides implications for developing future ESMSs that improve health-related outcomes for patients with cancer. Future research should focus on strengthening electronic equipment and technical support for ESMSs, improving their functional contents and participation forms, and developing personalized applications tailored to the specific needs and characteristics of patients with cancer.

**Trial Registration:**

PROSPERO CRD42023421730; https://www.crd.york.ac.uk/prospero/display_record.php?RecordID=421730

## Introduction

### Background

Globally, cancer incidence and mortality rates have increased rapidly, posing a major public health concern [1]. With the rapid development of cancer treatment methods, survival rates for patients with cancer have increased significantly [2,3]. However, the majority of patients with cancer experience numerous symptoms related to cancer and treatment-related toxicities [4-7], which may result in symptom distress [8,9], financial toxicity [10], prolonged hospitalization, high rates of complications, and even death [11].

Patient-reported outcomes (PROs) are defined as “measurements of any aspect of a patient's health status that come directly from the patient, without any interpretation by a clinician or anyone else” [12]. PROs have been shown to capture symptoms more accurately than physician assessments [13,14]. By using patients’ own assessments of the occurrence and severity of symptoms, health care professionals can identify and assess the potential health risks earlier, thereby improving patients’ health-related outcomes [15]. In recent years, there has been an explosion of paper versions of PROs, facilitated by a variety of validated instruments [13]. However, in real-world settings, paper scale assessments are prone to data loss and input errors, which make it difficult to guarantee data reliability and add to the burden of data management [16]. Additionally, when patients are not hospitalized, there is a lag in paper-based symptom assessment and management [17]. Given the limitations of paper-based symptom assessments, electronic methods for patients’ self-reported symptoms have shown significant promise [18].

Electronic symptom management systems (ESMSs) refer to electronic PRO (ePRO) systems that provide real-time patient assessment and symptom management in oncology practices. Based on patients’ responses to symptom assessments, ESMSs can automatically score symptoms and generate warnings. Afterward, health care professionals can receive the data and guide patients, and some ESMSs may also provide evidence-based symptom management recommendations [19-22]. Over the past decades, a growing number of ESMSs have been developed [23-25], and there is equivalence and comparability between electronic and paper-and-pencil symptom assessment measures [26]. Instead of relying on retrospective reporting and delayed manual documentation, ESMSs allow patients to report symptoms via their own electronic devices at home, as well as to potentially document these symptoms automatically in their medical records [27]. Additionally, by providing overviews of symptoms over time, ESMSs can also aid in the early detection and management of symptoms [28] and improve patient-clinician communication as well as patients’ quality of life [29-31].

In the past few years, the importance of ESMSs has become increasingly recognized by health care services. However, some studies found that patients’ engagement with ESMSs has declined over time [32-35]. Most current systematic reviews of ESMSs focus on intervention effectiveness [17,36] and identifying key mechanisms that improve patients’ health-related outcomes [37]. It must be noted that existing evidence fails to fully capture the details of patients’ profound experiences, and the underlying reasons for decreased engagement with ESMSs remain unclear. To overcome these deficiencies, systematic reviews of qualitative evidence can facilitate a better understanding of how patients perceive ESMSs. The existing systematic reviews of qualitative studies primarily emphasize patients’ experiences with telemedicine [38,39], including not only symptom monitoring and management but also telephone follow-up, digital consultation, virtual simulation, exercise intervention, and so on. However, this comprehensive coverage makes it difficult to probe deeply into patients’ specific experiences with ESMSs. Although numerous qualitative studies have been conducted to provide some insight into how patients with cancer perceive ESMSs, systematic reviews of qualitative studies that reflect the specific experiences of patients regarding ESMSs are lacking. Therefore, to better understand patients’ experiences with ESMSs, it is necessary to synthesize their experiences systematically.

### Objectives

This systematic review aims to synthesize previous qualitative studies on the experiences of patients with cancer using ESMSs, including their perceptions, preferences, barriers, and suggestions. This will contribute to the development of future ESMSs and electronic symptom management interventions tailored to patient needs, increasing the chances of patients reporting their symptoms in a timely manner, thereby resulting in positive outcomes both for patients and health care providers.

## Methods

### Overview

The protocol was registered in PROSPERO (CRD42023421730) prior to the systematic review. The systematic review was conducted in accordance with the PRISMA (Preferred Reporting Items for Systematic Reviews and Meta-Analyses) [40] statement checklist and the ENTREQ (Enhancing Transparency in Reporting the Synthesis of Qualitative Research) [41] statement to improve transparency in reporting the synthesis of qualitative research (Multimedia Appendices 1 and 2).

### Search Strategy

After assessing the relevant literature reviews, our trained reviewers (authors TF and YS) developed the search strategy and then carefully discussed it with the other reviewers to guarantee a systematic and comprehensive review of the papers. To develop our search strategy, keywords and derivatives of terms were identified via an initial, limited PubMed search. A combination of MeSH (Medical Subject Headings) terms and free terms were used to ensure all relevant papers were identified. The qualitative literature relevant to the study was searched using systematic electronic databases. The search strategy was developed for PubMed first and then adapted and applied to Web of Science, Cochrane Library, EBSCOhost, Embase, PsycINFO, ProQuest, Scopus, Wanfang database, CNKI, CBM, and VIP. We used the search terms “symptom manage*,” “symptom monitor*,” “mobile application*,” “cell phone,” “smartphone,” “internet,” “telemedicine,” “remote consultation,” “Computer-Assisted Decision Making,” “cancer,” “qualitative research,” and their MeSH terms. The full search strategy is shown in Multimedia Appendix 3. We searched the electronic databases for eligible studies from database inception to April 25, 2023, and updated it on January 2, 2024. The searches were conducted in English or Chinese. We used EndNote X9 (Clarivate) to upload and store the search results.

### Inclusion and Exclusion Criteria

Qualitative studies or qualitative components of mixed methods studies on the experiences of adult patients with cancer using ESMSs were included. Studies in which symptom monitoring and management were telephone-based or using passive monitoring were excluded. Textbox 1 describes the inclusion and exclusion criteria.

**Inclusion criteria and exclusion criteria.**

**Inclusion criteria**

- Patients diagnosed with cancer
- Aged 18 years and older
- Papers reporting on participants’ experiences with electronic symptom management systems (ESMSs) used to monitor and/or manage cancer-related symptoms
- Qualitative studies and the qualitative components of mixed methods research

**Exclusion criteria**

- Gray literature or unpublished peer-reviewed literature
- Published abstracts or conference proceedings
- Literature reviews, systematic reviews, meta-syntheses, etc
- Studies on patients with cancer with incurable illnesses or at the end of their lives
- Primary symptom monitoring and management that was telephone-based (eg, without using any internet-based health tools) or passive monitoring (eg, only using a wearable device)
- Papers published in languages other than English or Chinese

### Screening and Data Extraction

#### Data Screening

All data were imported into Endnote X9 software, and duplicates were removed. Two reviewers (authors SZ and YD) independently screened the papers based on the titles and abstracts. Subsequently, full-text publications that met the inclusion criteria were retrieved and screened by SZ and YD. Disagreements were resolved through discussion with other reviewers (authors YL, HW, XJ, MG, TF, YS, YZ, and YH) to reach a consensus.

#### Data Extraction

SZ and YD independently extracted the data using a standardized data extraction form (Multimedia Appendix 4). The data extraction focused on identifying specific qualitative results, such as the categories and subcategories related to the phenomenon of interest. For each study, descriptive data included information about the ESMSs, study objectives, methods, analyses, the country/region of study, and the participant demographics. Discrepancies were resolved through joint discussions with the other reviewers.

### Quality Appraisal

SZ and YD independently appraised all papers using the Critical Appraisal Skills Program (CASP) [42] criteria. The quality appraisal results were not used as exclusion criteria but helped determine the level of confidence in the findings. We were more concerned with papers that contained depth in data collection and analysis, which could provide valuable insight into participants’ experiences with ESMSs.

### Meta-Synthesis

The meta-synthesis method was used to synthesize the findings of the included studies. All authors read the included studies to understand the whole research. Three reviewers (authors SZ, YD, and HW) extracted findings that were closely related to our objectives, along with relevant quotations and authors’ interpretations. The quotations and interpretations were read and reread by SZ, YD, YL, and HW for coding. The codes were organized into subcategories to form categories. SZ, YD, and YL wrote and continuously refined draft summaries of the categories, and the review team evaluated the appropriateness of the synthesis. Any disagreements were resolved through team discussions.

## Results

### Summary of the Search Results

**Figure 1:**
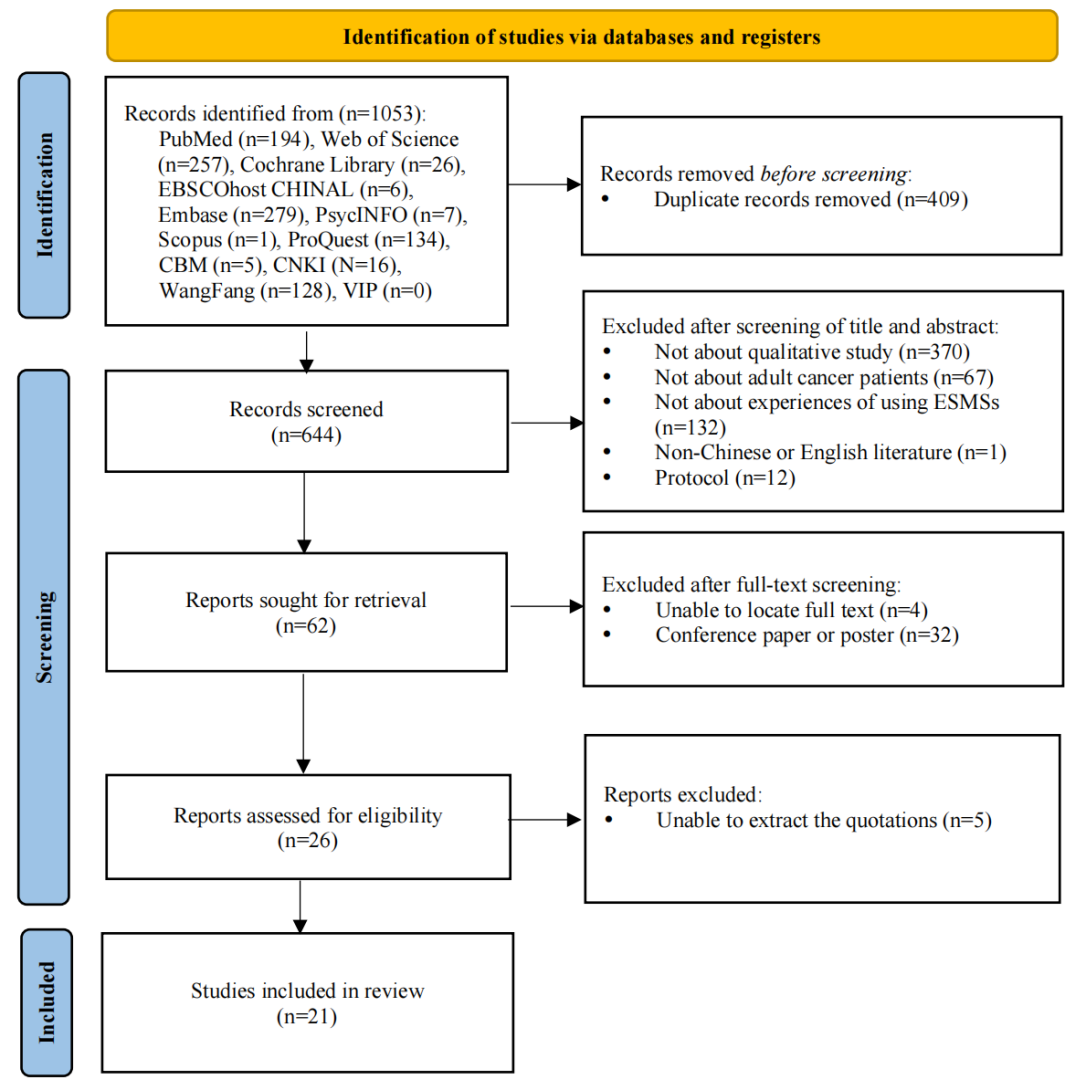
Study flow diagram adapted from the PRISMA (Preferred Reporting Items for Systematic Reviews and Meta-Analyses) flowchart. ESMS: electronic symptom management system.

Following the search strategy, 1053 papers were found. After the duplicate papers were removed, a total of 644 papers remained. After removing papers with irrelevant titles or abstracts, 62 papers were reviewed in full text. Ultimately, 21 papers were eligible for inclusion in the review. The PRISMA diagram (Figure 1) illustrates the results of the selection process.

The CASP scores of the papers ranged from 8.5 to 10 (Multimedia Appendix 5), indicating that all 21 papers met an acceptable level of quality and contributed to the meta-synthesis. The CASP results showed that in most included papers (n=20, 95.2%), the relationships between researchers and participants were not elaborated, thereby reducing the quality of the study.

The 21 included papers (Multimedia Appendix 6) were published between 2009 and 2023 and were conducted across diverse geographical settings, including the United Kingdom (n=5, 23.8%) [21,25,43-45], the United States (n=5, 23.8%) [24,46-49], Sweden (n=4, 19%) [23,30,31,50], Australia (n=2, 9.5%) [51,52], China (n=2, 9.5%) [53,54], Ireland (n=1, 4.8%) [20], Canada (n=1, 4.8%) [55], and Norway (n=1, 4.8%) [56]. There were 17 (81 %) mixed methods papers [21,23-25,30,31,43,45-47,49,51-56], while 4 (19%) were purely qualitative [20,44,48,50]. The sample sizes of the qualitative papers ranged from 3 to 131 participants, and 1 (4.8%) paper did not mention sample size [49]. In most (n=20, 95.2%) papers, patients with cancer were middle-aged or older adults. Most (n=15, 71.4%) papers included both male and female participants [20,21,23,25,44-46,48-52,54-56], whereas 2 (9.5%) papers only reported on males [30,31], 1 (4.8%) only reported on females [24], and 3 (14.3%) did not provide any information about gender at all [43,47,53]. In 5 (23.8%) papers [46,48,49,53,56], the cancer type was not examined, while others focused primarily on gastrointestinal cancer (n=7, 33.3%) [20,43-45,47,51,55], breast cancer (n=4, 19%) [23,24,43,51], prostate cancer (n=3, 14.3%) [23,30,31], lung cancer (n=3, 14.3%) [25,43,54], pancreatic cancer (n=1, 4.8%) [50], chronic myeloid leukemia (n=1, 4.8%) [52], lymphoma (n=1, 4.8%) [55], and malignant pleural mesothelioma (n=1, 4.8%) [21]. A total of 15 (71.4%) papers described treatments given to patients with cancer, including chemotherapy (n=5, 23.8%) [43,47,49,51,55], radiotherapy (n=4, 19%) [24,25,30,31], surgery (n=4, 19%) [44,45,48,50], and oral drugs (n=1, 4.8%) [52]. Moreover, 1 (4.8%) paper included patients receiving neoadjuvant chemotherapy for breast cancer and radiotherapy for prostate cancer [23].

In 8 (38.1%) papers, systematic symptom assessment scales were used in ESMSs, such as the Memorial Symptom Assessment Scale [20,23], Edmonton Symptom Assessment Scale [25], Rotterdam Symptom Checklist [51], Symptom Distress Scale [53], Pain-Intensity Numerical Scale [53], Modified Borg scale [54], Brief Fatigue Inventory [54], Brief Pain Inventory [54], Functional Assessment of Cancer Therapy [20,25,51], and European Organization for Research and Treatment of Cancer [44,45,53]. A total of 5 (23.8%) papers assessed psychological symptoms through ESMSs using the State-Trait Anxiety Inventory [20,25,54], Hospital Anxiety and Depression Scale [51], and Patient Health Questionnaire-Depression Model [53]. In terms of ESMS use time, participants in 15 (71.4%) papers used ESMSs for periods ranging from 10 days to 18 weeks [20,21,23-25,30,31,43-45,47,48,50-52], and most assessed symptoms daily (n=9, 42.9%) [20,21,23,25,30,31,48,50,51]. Additionally, 3 (14.3%) papers were conducted to assess the usability of ESMSs at specific sites [46,55,56], 2 (9.5%) of which required participants to self-report symptoms while waiting in the hospital [53,54], and 1 (4.8%) at the next 2 scheduled chemotherapy appointments [49].

We found that ESMSs varied in the form of symptom management. For the majority of ESMSs mentioned, patients were provided with symptom assessment (n=21, 100%), alerts regarding symptoms were sent to health care professionals (n=12, 57.1%) [21,23-25,30,31,43-45,48,50,55], health care professionals responded to symptoms (n=10, 47.6%) [20,21,23-25,31,43,48,50,55], symptom self-care advices were provided to patients (n=18, 85.7%) [20,21,23,25,30,31,43-48,50-52,54-56], and symptom histories were presented as graphs over time (n=9, 42.9%) [21,23,30,31,44,45,50,51,56]. Furthermore, ESMSs were also capable of allowing patients to create favorites [46], write free text for private health-related information [56], receive daily medication reminders [52], exchange messages with health care professionals [46,56], and share information and experiences with other patients [56].

### Results of the Meta-Synthesis

#### Overview

Participants' experiences with ESMSs were categorized into 3 major categories and 10 subcategories. Multimedia Appendix 7 includes detailed quotations from each of the 3 major categories and 10 subcategories.

#### Category 1: Perceptions and Attitudes Toward ESMSs

##### Category Overview

This category demonstrated participants’ positive and negative perceptions and attitudes toward ESMSs. The participants reported that ESMSs were easy to use, supported symptom monitoring and management, and became a part of their daily routines, but they also expressed some negative perspectives.

##### Subcategory 1: Ease of Use

In 13 (61.9%) papers, the participants praised the ease of use of ESMSs [20,21,25,30,43,46-48,50,51,54-56]. In general, participants found ESMSs to be easy to use and navigate [21,25,30,43,46-48]. Participants who were not accustomed to using ESMSs experienced troubles at first, but once they were trained or used them a few times, were able to use them smoothly [50,51,55,56]. A number of participants appreciated how ESMSs asked questions regarding symptoms in lay terms that were easy to understand [20]. Moreover, compared with pen-and-paper formats and face-to-face assessments, the participants found ESMSs more convenient since they could complete the questionnaire at their own pace and correct any incorrect responses themselves [50,54]. Additionally, ESMS also enabled participants to gain a better understanding and mastery of self-management information by providing health information in an accessible format [54].

##### Subcategory 2: Support Symptom Monitoring and Management

The role of ESMSs in supporting symptom monitoring and management was discussed in 14 (66.7%) papers [20,23,25,31,44-53]. Participants highly appreciated that ESMSs enabled and prompted them to describe symptoms accurately [20,49,50,53]. Some participants believed that ESMSs helped them recall symptoms in general [20,49] and “were good at telling them what’s normal or not” [45]. In addition, the participants felt that their symptoms were being tracked by someone [20,46,48,51,52], and the symptoms graphs in ESMSs helped them become aware of and track their symptoms over time [23,44,45,50,51]. Moreover, the participants found ESMSs helpful for reinforcing medical guidance they had received and reminding them of any information they had forgotten [25,44,45,51]. The participants also appreciated that the self-care information provided by ESMSs was specific, appropriate, and achievable, which helped them cope better with their symptoms [20,31,44,45,47,50,51].

##### Subcategory 3: Symptom Reporting Becomes a Part of Their Daily Routines

As described in 3 (14.3%) papers, the daily requirement of ESMSs to complete the symptom questionnaire became embedded in participants’ daily routines [20,30,43]. Although it took a little time to complete the symptom assessment, this did not negatively impact participants’ daily lives, encouraging them to establish a routine.

##### Subcategory 4: Negative Perspectives

A total of 5 (23.8%) papers reported that participants with negative perspectives regarding ESMSs were less motivated to use them [24,25,30,53,54]. A few participants questioned the clinical relevance of ESMSs [24]. Several participants expressed concern that their symptom reports would be ignored, and if so, they would perceive using ESMSs as a waste of time and worthless [53]. Some participants felt disappointed at not being contacted and doubted the trustworthiness of ESMSs [30]. Some participants considered face-to-face communication to be more reliable and professional than communication through ESMSs [54], and some expressed concern about the additional workload and pressure added to doctors to digest so much information simultaneously [53]. Furthermore, some participants perceived that the self-care information in ESMSs was similar to what their health care providers provided, so they had never or only occasionally read it [25].

#### Category 2: The Value of ESMSs

##### Category Overview

As shown in this category, ESMSs were found to be valuable for the participants. The ESMSs helped motivate participants to monitor and manage their symptoms, connect with one another, and communicate effectively, thereby promoting psychological well-being. Despite rarely being mentioned, the participants recognized the potential benefits of ESMSs in alleviating health care burdens.

##### Subcategory 1: Increasing Motivation for Symptom Monitoring and Management

A total of 9 (42.9%) papers found that ESMSs improved participants’ motivation for self-symptom monitoring and management [20,24,30,48-52,54]. ESMSs enhanced the participants’ understanding of the causes and effects of symptoms [24,30,49,50,54] and stimulated their consideration of effective methods for symptom prevention and management [20,48,49,51,52]. Moreover, both newly diagnosed and long-term participants reported that using ESMSs improved their adherence by strengthening their sense of accountability for monitoring and managing their symptoms [52].

##### Subcategory 2: Enhancing Connection and Effective Communication With Others

The benefits of ESMSs in facilitating participants’ communication with their health care providers, families, and other patients were reported in 15 (71.4%) papers [20,21,25,30,31,43,44,48-53,55,56]. Participants did not feel that their connection with health care providers was interrupted by using ESMSs, and they were able to maintain a sense of connection after returning home [20,44,50]. Moreover, it was noted that ESMSs’ data collection capabilities and alerting mechanisms provided participants with quick access to health care providers [20,25,30,31,43,48,51,55] and could increase their chances of receiving further consultations and treatment [20,21,30,31,44]. Additionally, the ESMSs helped participants prepare for consultations before their visits [49], saving time during medical consultations [53] and facilitating effective communication with health care providers [49,52,53]. The participants also described how ESMS graphs helped them share their feelings and symptoms with their families and friends [30]. Finally, the ESMSs helped facilitate communication and cooperation between patients [56].

##### Subcategory 3: Gaining Positive Psychological Experiences

A total of 13 (61.9%) papers reported the psychological benefits associated with using ESMSs [20,21,23,25,31,43-45,49-52]. Participants appreciated how ESMSs made them feel reassured, regardless of whether they were experiencing symptoms or not [20,21,23,24,31,43-45,50,51]. The participants also appreciated how ESMSs helped them overcome feelings of uncertainty and concern about symptoms [20,25,45] and reduced anxiety and nervousness [20,44,49]. Interestingly, some participants felt they were being listened to and cared for when using ESMSs [21,52].

##### Subcategory 4: Potential Benefits for Health Care

As noted in 2 (9.5%) of the reviewed papers [30,43], ESMS use may benefit both patients with cancer and health care professionals. Patients believed that ESMSs would reduce the frequency of their contacting hospitals with symptoms or health-related concerns, thus saving them time spent on phone consultations [43]. Additionally, ESMSs could reduce the burden on the health care system as some patients view them as similar to having medical staff at home [30].

#### Category 3: Barriers, Requirements and Suggestions for ESMSs

##### Category Overview

Although most participants reported that ESMSs were easy to operate, several barriers remained. In terms of functionality and content, the participants emphasized the importance of questionnaire items, reminders, alerts, and health information. They also provided suggestions for interface settings based on their needs.

##### Subcategory 1: Barriers to Using ESMSs

In 6 (28.6%) papers, barriers to using ESMSs were mentioned [24,51-54,56]. Participants who were older or had a lower educational level reported difficulties understanding the questionnaire items and using mobile devices [24,53,54,56]. Participants’ health conditions related to cancer treatments are also one of the barriers to using ESMSs, such as being “too tired to follow it through” [51] or worrying about being “too physically or psychologically unwell to participate in the assessment” [53]. Additionally, rural residents, even those who could use mobile devices at home, encountered problems transmitting symptom assessment information to clinical centers due to unstable cellular or internet connectivity [52].

##### Subcategory 2: Requirements and Suggestions for ESMSs

A total of 12 (57.1%) papers highlighted the requirements and suggestions for ESMSs’ symptom assessment, symptom alerts, information push, and interface settings [23,24,30,43-46,48,50,51,53,55]. Participants expressed a desire for improvements in the simplicity and accuracy of the symptom assessment items [24,30,53], as well as the ability to report additional symptoms [24,43,48]. Moreover, participants with varying disease characteristics had significantly different needs for symptom assessment frequency, ranging from twice a day to once every 6 months [23,24,43,51,53]. Participants also mentioned that they sometimes forgot to log in and record symptoms [51], so some suggested adding an alert to remind them [43]. Furthermore, the option of entering data retrospectively and reediting previously entered data was recommended by participants [51].

While symptom alerts varied from study to study, many participants expressed a desire to decide when or whether to be contacted or to contact health care providers on their own [30,43-45]. Some participants did not consider their symptoms serious enough to warrant consultation [50]. A few even described how they had learned to adjust their responses to avoid being called by nurses [30]. Additionally, some participants did not contact health care providers because their symptoms had already been treated [45] or appointments had already been scheduled [44,45].

Regarding symptom management information push, some participants wanted instant feedback on their symptoms after completing the assessment, as they viewed their symptom results as their personal property [53]. They also hoped that ESMSs would offer better categorized, personalized self-care information [55] and provide different daily messages rather than the same one every day [51]. In addition, having access to health information tailored to their conditions, as well as information on specific topics, was also important to them [51].

In terms of user interface settings, participants reported that they were more likely to use ESMSs with a visually appealing and advanced user interface [46,53,55]. They recommended adding a higher-level menu and a search button to simplify finding information and save time [55]. A larger screen or larger text was considered beneficial for participants with poor eyesight [55]. Additionally, some participants commented that “the screen seemed to be too monotonous” or “clinical” [46,53] and suggested making it more colorful or vivid to attract users’ attention [53,55].

## Discussion

### Principal Findings

This qualitative systematic review indicated that patients with cancer had both positive and negative experiences with ESMSs. In addition to their ease of use, usefulness, and convenience, patients with cancer benefited from ESMSs physically, psychologically, socially, and economically. However, some patients with cancer were discouraged from using ESMSs due to negative perceptions and barriers. Furthermore, patients with cancer require ESMSs that could be tailored to their specific needs.

This review indicates that ESMSs are useful and acceptable. Consistent with other studies [36,57,58], ESMSs are easy to use, help patients with cancer accurately describe and continuously track their symptoms, provide them with practical self-care information, and do not interfere with their daily lives. Nevertheless, similar to other relevant reviews [59], we found that some patients with cancer had negative experiences with ESMSs, which affected their usage intentions.

Our study indicated that communication with health care providers via mobile phones was not considered more reliable than face-to-face communication. This may be because patients do not want to change their offline medical habits, and they believe using traditional face-to-face forms would help build trust and facilitate better communication [59]. Furthermore, previous studies have shown that the stickiness of ESMSs has decreased over time among patients [32-35], and the reasons behind this decline were explored in our study. Patients with cancer may lose motivation and willingness to continue using ESMSs if they do not see their value, discover that their symptom management information is similar to that provided by their health care providers, or are not provided with timely feedback on their self-reports. According to the Technology Acceptance Model [60], perceived usefulness is one of the key factors affecting users’ attitudes and behavior toward new technologies. In this way, patients with cancer are more likely to use and adhere to ESMSs when they perceive that the ESMSs can aid them with symptom management.

This study further confirms the value of ESMSs in real-world applications. As demonstrated in previous telemedicine studies [38,61], ESMSs can increase patient awareness of and attention to symptoms monitoring and management, facilitate effective communication with health care professionals and other patients, and enhance positive psychological experiences. This study also found long-term potential benefits of ESMSs in reducing health care burdens [30,43]. According to Jibb et al [62], although following up with the health care team after alerts increases intervention costs, it can reduce the rate of emergency department visits, prevent patients from missing the optimal treatment time during diagnosis and treatment queues, and lower overall medical costs. Additionally, Zhang et al [63] found that ESMSs can collect patient symptom information in a timely and continuous manner, thus reducing the cost of manual information collection.

While patients with cancer benefited from the ESMSs, some still encountered barriers to their use. Like other studies regarding technology use in medical settings, patients who were older [59,64], less educated [65,66], and lived in rural areas [67] faced greater challenges with using ESMSs. Older adults tend to have high levels of technology anxiety and even resistance to using technology [68], possibly resulting in a digital divide that hinders their use of ESMSs [69]. Moreover, less educated patients may have difficulty understanding and using the complex functions of ESMSs [38], and patients in rural areas may lack adequate infrastructure and technical support [38]. Due to limited access to network and technological resources, and limited information literacy, mobile medical treatments are challenging for these vulnerable groups [38,70].

This study also provided useful insights into how ESMSs can be further developed and optimized. Patients with cancer have a variety of needs regarding the content, form, and other aspects of ESMSs, and whether these needs are met may affect their adoption and subsequent use [71]. These findings suggest that ESMSs should be improved in terms of functionality and content design to better meet patients’ needs. Consideration should be given to reducing the burden of completing symptom assessments, adjusting symptom alert settings based on whether patients need assistance, and providing more personalized symptom management information. Furthermore, it is imperative to add symptom-filling reminders, modifications, and feedback functions and to create a user-friendly interface.

### Strengths and Limitations

To the best of our knowledge, this is the first meta-synthesis focusing on the experiences of patients with cancer who used ESMSs for symptom monitoring and management. This review covered all existing ESMSs regardless of the cancer type, providing a better understanding of how patients with cancer perceive them. Thus, it may provide guidance for future ESMS development and optimization.

Despite the significant insights provided by this review, it faced some limitations. First, this systematic review was limited to articles published in English or Chinese due to the language restrictions of the research group. Although some studies were conducted in non-English speaking areas, they can still result in biased results and be missed. Second, since people with negative attitudes toward ESMSs are likely to be excluded from research studies, this review may not fully reflect all the experiences of patients with cancer. Finally, most of the included studies were from Europe and America and may not be representative of patients with cancer from other regions.

### Recommendations for the Future

Based on our meta-synthesis, we have come up with the following recommendations for the future. In the process of using ESMSs, it is important to pay attention to the plight of vulnerable patient groups and provide them with necessary assistance in adapting to this system. Furthermore, future studies should explore what resources patients on different cancer journeys require and prefer rather than simply assuming and prescribing what is deemed beneficial and useful. Additionally, developing ESMSs requires the participation of a multidisciplinary team to bridge the gap between technological innovation and successful service delivery.

### Conclusions

This study provides an overview of the qualitative literature on the experiences of patients with cancer using ESMSs. Overall, the review indicated that ESMSs resulted in positive symptom monitoring and management experiences for patients. However, some patients encountered barriers during the use process, so their individual needs and preferences must be carefully considered. Recommendations from this systematic review can be used to improve ESMS development, adoption, and compliance.

## Appendix

Multimedia Appendix 1 PRISMA (Preferred Reporting Items for Systematic Reviews and Meta-Analyses) 2020 checklist.

Multimedia Appendix 2 ENTREQ (Enhancing Transparency in Reporting the Synthesis of Qualitative Research) checklist.

Multimedia Appendix 3 Search strategy.

Multimedia Appendix 4 Extracted data form.

Multimedia Appendix 5 Critical Appraisal Skills Program (CASP) quality appraisal of the included studies.

Multimedia Appendix 6 Sample characteristics of the included studies.

Multimedia Appendix 7 Synthesis of the findings.

## Abbreviations

CASP: Critical Appraisal Skills Program
ENTREQ: Enhancing Transparency in Reporting the Synthesis of Qualitative Research
ePRO: electronic patient-report outcome
ESMS: electronic symptom management system
MeSH: Medical Subject Headings
PRISMA: Preferred Reporting Items for Systematic Reviews and Meta-Analyses
PRO: patient-reported outcome
