# Supplementary material for: Experiences of Patients With Cancer Using Electronic Symptom Management Systems: Qualitative Systematic Review and Meta-Synthesis
Source: J Med Internet Res. 2024 Oct 28;26:e59061. doi: 10.2196/59061 (PMC11555449; doi:10.2196/59061)
Supplement: Multimedia Appendix 3 [file jmir_v26i1e59061_app3.docx]

| **1. Search strategy for PubMed** | |
| --- | --- |
| #1 | “symptom manage*”[Title/Abstract] OR “symptom self manage*”[Title/Abstract] OR “management of symptom*”[Title/Abstract] OR “symptom monitor*”[Title/Abstract] |
| #2 | “Mobile Applications”[Mesh] OR “mobile application*”[Title/Abstract] OR mobile*[Title/Abstract] OR app[Title/Abstract] OR apps[Title/Abstract] OR “health application*”[Title/Abstract] OR “mobile technolog*”[Title/Abstract] |
| #3 | “Cell Phone”[Mesh] OR “Cell Phone Use”[Mesh] OR phone*[Title/Abstract] OR telephone*[Title/Abstract] |
| #4 | “Smartphone”[Mesh] OR smartphone*[Title/Abstract] OR “smart phone*” [Title/Abstract] OR “smart device*”[Title/Abstract] |
| #5 | “Internet”[Mesh] OR internet*[Title/Abstract] OR “world wide web*”[Title/Abstract] OR “web-based”[Title/Abstract] OR “web based”[Title/Abstract] OR “web-delivered”[Title/Abstract] OR “internet delivered”[Title/Abstract] OR “internet supported”[Title/Abstract] OR “internet mediat*”[Title/Abstract] OR “internet-based”[Title/Abstract] OR “web page*”[Title/Abstract] OR “web application*”[Title/Abstract] |
| #6 | “Telemedicine”[Mesh] OR “Virtual Medicine”[Title/Abstract] OR telemedic*[Title/Abstract] OR telehealth*[Title/Abstract] OR “tele-medic*”[Title/Abstract] OR “tele-health*”[Title/Abstract] OR telemonitor*[Title/Abstract] OR “tele-monitor*”[Title/Abstract] OR ehealth*[Title/Abstract] OR “e-health*”[Title/Abstract] OR “electric health*”[Title/Abstract] OR “m-health*”[Title/Abstract] OR mhealth*[Title/Abstract] OR “mobile health*”[Title/Abstract] OR “e-therap*”[Title/Abstract] OR etherap*[Title/Abstract] OR “electronic pro”[Title/Abstract] OR “electronic prom”[Title/Abstract] OR epro[Title/Abstract] OR eprom[Title/Abstract] OR “e-pro”[Title/Abstract] OR “e-prom”[Title/Abstract] OR “electronic patient reported outcome*”[Title/Abstract] OR “digital*”[Title/Abstract] OR “intelligen*”[Title/Abstract] |
| #7 | “Remote Consultation”[Mesh] OR teleconsultation*[Title/Abstract] OR “remote monitor*”[Title/Abstract] OR “remotely monitor*”[Title/Abstract] |
| #8 | “Decision Making, Computer-Assisted”[Mesh] OR “Decision Making, Computer Assisted”[Title/Abstract] OR “Computer-Assisted Decision Making”[Title/Abstract] OR “Computer Assisted Decision Making”[Title/Abstract] OR “Medical Decision Making, Computer-Assisted”[Title/Abstract] OR “Medical Decision Making, Computer Assisted” [Title/Abstract] |
| #9 | #2 OR #3 OR #4 OR #5 OR #6 OR #7 OR #8 |
| #10 | “Neoplasms”[Mesh] OR Tumor*[Title/Abstract] OR Neoplas*[Title/Abstract] OR Cancer*[Title/Abstract] OR Malignan*[Title/Abstract] OR carcinoma*[Title/Abstract] |
| #11 | “Qualitative Research”[Mesh] OR qualitative research*[Title/Abstract] OR qualitative stud*[Title/Abstract] OR qualitative method*[Title/Abstract] OR mixed research*[Title/Abstract] OR mixed stud*[Title/Abstract] OR mixed method*[Title/Abstract] OR case stud*[Title/Abstract] OR constant comparis*[Title/Abstract] OR content analy*[Title/Abstract] OR descriptive stud*[Title/Abstract] OR phenomeno* [Title/Abstract] OR grounded theor*[Title/Abstract] OR focus group* [Title/Abstract] OR ethnography[Title/Abstract] OR narrative*[Title/Abstract] OR interview*[Title/Abstract] OR action research*[Title/Abstract] OR participant observ*[Title/Abstract] OR thematic analy*[Title/Abstract] OR feminist[Title/Abstract] OR naturalistic[Title/Abstract] |
| #12 | #1 AND #9 AND #10 AND #11 |
| **2. Search strategy for Web of Science** | |
| #1 | TS=("symptom manage*" OR "symptom self manage*" OR "management of symptom*" OR "symptom monitor*") |
| #2 | TS=("Mobile Applications" OR “mobile application*” OR mobile* OR app OR apps OR “health application*” OR “mobile technolog*”) |
| #3 | TS=("Cell Phone" OR “Cell Phone Use" OR phone* OR telephone*) |
| #4 | TS=("Smartphone" OR smartphone* OR “smart phone*” OR “smart device*”) |
| #5 | TS=(“Internet” OR internet* OR “world wide web*” OR “web-based” OR “web based” OR “web-delivered” OR “internet delivered” OR “internet supported” OR “internet mediat*” OR “internet-based” OR “web page*” OR “web application*”) |
| #6 | TS=("Telemedicine" OR "Virtual Medicine" OR telemedic* OR telehealth* OR “tele-medic*” OR “tele-health*” OR telemonitor* OR “tele-monitor*” OR ehealth* OR “e-health*” OR “electric health*” OR “m-health*” OR mhealth* OR “mobile health*” OR “e-therap*” OR etherap* OR “electronic pro” OR “electronic prom” OR epro OR eprom OR “e-pro” OR “e-prom” OR “electronic patient reported outcome*” OR “digital*” OR “intelligen*”) |
| #7 | TS=(“Remote Consultation” OR teleconsultation* OR “remote monitor*” OR “remotely monitor*”) |
| #8 | TS=("Decision Making, Computer-Assisted" OR “Decision Making, Computer Assisted” OR “Computer-Assisted Decision Making” OR “Computer Assisted Decision Making” OR “Medical Decision Making, Computer-Assisted” OR “Medical Decision Making, Computer Assisted”) |
| #9 | #2 OR #3 OR #4 OR #5 OR #6 OR #7 OR #8 |
| #10 | TS=("Neoplasms" OR Tumor* OR Neoplas* OR Cancer* OR Malignan* OR carcinoma*) |
| #11 | TS=("Qualitative Research" OR qualitative research* OR qualitative stud* OR qualitative method* OR mixed research* OR mixed stud* OR mixed method* OR case stud* OR constant comparis* OR content analy* OR descriptive stud* OR phenomeno* OR grounded theor* OR focus group* OR ethnography OR narrative* OR interview* OR action research* OR participant observ* OR thematic analy* OR feminist OR naturalistic) |
| #12 | #1 AND #9 AND #10 AND #11 |
| **3. Search strategy for Cochrane Library** | |
| #1 | (symptom management):ti,ab,kw OR (symptom self management):ti,ab,kw OR (management of symptom):ti,ab,kw OR (symptom monitor):ti,ab,kw |
| #2 | MeSH descriptor: [Mobile Applications] explode all trees |
| #3 | MeSH descriptor: [Smartphone] explode all trees |
| #4 | MeSH descriptor: [Internet] explode all trees |
| #5 | MeSH descriptor: [Telemedicine] explode all trees |
| #6 | MeSH descriptor: [Decision Making, Computer-Assisted] explode all trees |
| #7 | (mobile*):ti,ab,kw OR (app):ti,ab,kw OR (apps):ti,ab,kw OR (“health application”):ti,ab,kw OR (“mobile technology”):ti,ab,kw |
| #8 | (cell phone):ti,ab,kw OR (phone*):ti,ab,kw OR (telephone*):ti,ab,kw OR ("smart phone"):ti,ab,kw OR ("smart device"):ti,ab,kw |
| #9 | (“world wide web”):ti,ab,kw OR (“web-based”):ti,ab,kw OR (“web-delivered”):ti,ab,kw OR (“internet delivered”):ti,ab,kw OR (“internet supported”):ti,ab,kw |
| #10 | (“internet-based”):ti,ab,kw OR (“web application”):ti,ab,kw OR ("Virtual Medicine"):ti,ab,kw OR (telehealth*):ti,ab,kw OR (telemonitor*):ti,ab,kw |
| #11 | (ehealth*):ti,ab,kw OR (“electric health”):ti,ab,kw OR (mhealth*):ti,ab,kw OR (“mobile health”):ti,ab,kw OR (etherap*):ti,ab,kw |
| #12 | (“electronic pro”):ti,ab,kw OR (“electronic prom”):ti,ab,kw OR (epro):ti,ab,kw OR (eprom):ti,ab,kw OR (“electronic patient reported outcome”):ti,ab,kw |
| #13 | (digital*):ti,ab,kw OR (intelligen*):ti,ab,kw OR (“Remote Consultation”):ti,ab,kw OR (teleconsultation*):ti,ab,kw OR (“remote monitor”):ti,ab,kw |
| #14 | (“Computer-Assisted Decision Making”):ti,ab,kw OR (“Computer Assisted Decision Making”):ti,ab,kw |
| #15 | #2 OR #3 OR #4 OR #5 OR #6 OR #7 OR #8 OR #9 OR #10 OR #11 OR #12 OR #13 OR #14 |
| #16 | MeSH descriptor: [Neoplasms] explode all trees |
| #17 | (tumor*):ti,ab,kw OR (cancer*):ti,ab,kw OR (malignan*):ti,ab,kw OR (carcinoma*):ti,ab,kw |
| #18 | #16 OR #17 |
| #19 | MeSH descriptor: [Qualitative Research] explode all trees |
| #20 | ("qualitative study"):ti,ab,kw OR ("qualitative method"):ti,ab,kw OR ("mixed research"):ti,ab,kw OR ("mixed study"):ti,ab,kw OR ("mixed method"):ti,ab,kw |
| #21 | ("case study"):ti,ab,kw OR ("constant conparison"):ti,ab,kw OR ("content analysis"):ti,ab,kw OR ("descriptive study"):ti,ab,kw OR (phenomenology):ti,ab,kw |
| #22 | ("grounded theory"):ti,ab,kw OR ("focus group"):ti,ab,kw OR (ethnography):ti,ab,kw OR ("narrative theory"):ti,ab,kw OR ("interview study"):ti,ab,kw |
| #23 | ("action research"):ti,ab,kw OR ("participant observation"):ti,ab,kw OR ("thematic analysis"):ti,ab,kw OR (feminist):ti,ab,kw OR (naturalistic):ti,ab,kw |
| #24 | #19 OR #20 OR #21 OR #22 OR #23 |
| #25 | #1 AND #15 AND #18 AND #24 |
| **4. Search Strategy for EBSCOhost CHINAL** | |
| #1 | SU=("symptom manage*" OR "symptom self manage*" OR "management of symptom*" OR "symptom monitor*") |
| #2 | SU=("Mobile Applications" OR "mobile application*" OR mobile* OR app OR apps OR "health application*" OR "mobile technolog*" OR "Cell Phone" OR "Cell Phone Use" OR phone* OR telephone* OR "Smartphone" OR smartphone* OR “smart phone*” OR “smart device*” OR “Internet” OR internet* OR “world wide web*” OR “web-based” OR “web based” OR “web-delivered” OR “internet delivered” OR “internet supported” OR “internet mediat*” OR “internet-based” OR “web page*” OR “web application*” OR "Telemedicine" OR "Virtual Medicine" OR telemedic* OR telehealth* OR “tele-medic*” OR “tele-health*” OR telemonitor* OR “tele-monitor*” OR ehealth* OR “e-health*” OR “electric health*” OR “m-health*” OR mhealth* OR “mobile health*” OR “e-therap*” OR etherap* OR “electronic pro” OR “electronic prom” OR epro OR eprom OR “e-pro” OR “e-prom” OR “electronic patient reported outcome*” OR “digital*” OR “intelligen*” OR “Remote Consultation” OR teleconsultation* OR “remote monitor*” OR “remotely monitor*” OR "Decision Making, Computer-Assisted" OR “Decision Making, Computer Assisted” OR “Computer-Assisted Decision Making” OR “Computer Assisted Decision Making” OR “Medical Decision Making, Computer-Assisted” OR “Medical Decision Making, Computer Assisted”) |
| #3 | SU=("Neoplasms" OR Tumor* OR Neoplas* OR Cancer* OR Malignan* OR carcinoma*) |
| #4 | SU=("Qualitative Research" OR qualitative research* OR qualitative stud* OR qualitative method* OR mixed research* OR mixed stud* OR mixed method* OR case stud* OR constant comparis* OR content analy* OR descriptive stud* OR phenomeno* OR grounded theor* OR focus group* OR ethnography OR narrative* OR interview* OR action research* OR participant observ* OR thematic analy* OR feminist OR naturalistic) |
| #5 | #1 AND #2 AND #3 AND #4 |
| **5. Search Strategy for Embase** | |
| #1 | 'symptom manage*':ti,ab,kw OR 'symptom self manage*':ti,ab,kw OR 'management of symptom*':ti,ab,kw OR 'symptom monitor*':ti,ab,kw |
| #2 | 'mobile phone'/exp |
| #3 | 'smartphone'/exp |
| #4 | 'internet'/exp |
| #5 | 'telemedicine'/exp |
| #6 | 'teleconsultation'/exp |
| #7 | 'decision support system'/exp |
| #8 | 'health application*':ti,ab,kw OR 'mobile technolog*':ti,ab,kw OR 'cell phone':ti,ab,kw OR phone*:ti,ab,kw OR telephone*:ti,ab,kw OR smartphone*:ti,ab,kw OR 'smart phone*':ti,ab,kw OR 'smart device*' OR internet*:ti,ab,kw OR 'world wide web*':ti,ab,kw OR 'web-based':ti,ab,kw OR 'web based':ti,ab,kw OR 'web-delivered':ti,ab,kw OR 'internet delivered':ti,ab,kw OR 'internet supported':ti,ab,kw OR 'internet mediat*':ti,ab,kw OR 'internet-based':ti,ab,kw OR 'web page*':ti,ab,kw OR 'web application*':ti,ab,kw OR 'virtual medicine':ti,ab,kw OR telemedic*:ti,ab,kw OR telehealth*:ti,ab,kw OR 'tele-medic*':ti,ab,kw OR 'tele-health*':ti,ab,kw OR telemonitor*:ti,ab,kw OR 'tele-monitor*':ti,ab,kw OR ehealth*:ti,ab,kw OR 'e-health*':ti,ab,kw OR 'electric health*':ti,ab,kw OR 'm-health*':ti,ab,kw OR mhealth*:ti,ab,kw OR 'mobile health*':ti,ab,kw OR 'e-therap*':ti,ab,kw OR etherap*:ti,ab,kw OR 'electronic pro':ti,ab,kw OR 'electronic prom':ti,ab,kw OR epro:ti,ab,kw OR eprom:ti,ab,kw OR 'e-pro':ti,ab,kw OR 'e-prom':ti,ab,kw OR 'electronic patient reported outcome*':ti,ab,kw OR 'digital*':ti,ab,kw OR intelligen*:ti,ab,kw OR 'remote consultation':ti,ab,kw OR teleconsultation*:ti,ab,kw OR 'remote monitor*':ti,ab,kw OR 'remotely monitor*':ti,ab,kw OR 'decision making, computer-assisted':ti,ab,kw OR 'decision making, computer assisted':ti,ab,kw OR 'computer-assisted decision making':ti,ab,kw OR 'computer assisted decision making':ti,ab,kw OR 'medical decision making, computer-assisted':ti,ab,kw OR 'medical decision making, computer assisted':ti,ab,kw |
| #9 | #2 OR #3 OR #4 OR #5 OR #6 OR #7 OR #8 |
| #10 | 'malignant neoplasm'/exp |
| #11 | 'tumor*':ti,ab,kw OR 'neoplas*':ti,ab,kw OR 'cancer*':ti,ab,kw OR 'malignan*':ti,ab,kw OR 'carcinoma*' |
| #12 | #10 OR #11 |
| #13 | 'qualitative research'/exp |
| #14 | 'qualitative study':ti,ab,kw OR 'qualitative method':ti,ab,kw OR 'mixed research':ti,ab,kw OR 'mixed study':ti,ab,kw OR 'mixed method':ti,ab,kw OR 'case study':ti,ab,kw OR 'constant comparision':ti,ab,kw OR 'content analysis':ti,ab,kw OR 'descriptive study':ti,ab,kw OR 'phenomenology':ti,ab,kw OR 'grounded theory':ti,ab,kw OR 'focus group':ti,ab,kw OR 'ethnography':ti,ab,kw OR 'narrative':ti,ab,kw OR 'interview*':ti,ab,kw OR 'action research':ti,ab,kw OR 'participant observation':ti,ab,kw OR 'thematic analysis':ti,ab,kw OR 'feminist':ti,ab,kw OR 'naturalistic':ti,ab,kw |
| #15 | #13 OR #14 |
| #16 | #1 AND #9 AND #12 AND #15 |
| **6. Search Strategy for PsycINFO** | |
| #1 | SU ("symptom manage*" OR "symptom self manage*" OR "management of symptom*" OR "symptom monitor*") |
| #2 | SU ("Mobile Applications" OR “mobile application*” OR mobile* OR app OR apps OR “health application*” OR “mobile technolog*” OR "Cell Phone" OR “Cell Phone Use" OR phone* OR telephone* OR "Smartphone" OR smartphone* OR “smart phone*” OR “smart device*” OR “Internet” OR internet* OR “world wide web*” OR “web-based” OR “web based” OR “web-delivered” OR “internet delivered” OR “internet supported” OR “internet mediat*” OR “internet-based” OR “web page*” OR “web application*” OR "Telemedicine" OR "Virtual Medicine" OR telemedic* OR telehealth* OR “tele-medic*” OR “tele-health*” OR telemonitor* OR “tele-monitor*” OR ehealth* OR “e-health*” OR “electric health*” OR “m-health*” OR mhealth* OR “mobile health*” OR “e-therap*” OR etherap* OR “electronic pro” OR “electronic prom” OR epro OR eprom OR “e-pro” OR “e-prom” OR “electronic patient reported outcome*” OR “digital*” OR “intelligen*” OR “Remote Consultation” OR teleconsultation* OR “remote monitor*” OR “remotely monitor*” OR "Decision Making, Computer-Assisted" OR “Decision Making, Computer Assisted” OR “Computer-Assisted Decision Making” OR “Computer Assisted Decision Making” OR “Medical Decision Making, Computer-Assisted” OR “Medical Decision Making, Computer Assisted”) |
| #3 | SU ("Neoplasms" OR Tumor* OR Neoplas* OR Cancer* OR Malignan* OR carcinoma*) |
| #4 | SU ("Qualitative Research" OR qualitative research* OR qualitative stud* OR qualitative method* OR mixed research* OR mixed stud* OR mixed method* OR case stud* OR constant comparis* OR content analy* OR descriptive stud* OR phenomeno* OR grounded theor* OR focus group* OR ethnography OR narrative* OR interview* OR action research* OR participant observ* OR thematic analy* OR feminist OR naturalistic) |
| #5 | #1 AND #2 AND #3 AND #4 |
| **7. Search Strategy for Scopus** | |
| #1 | TITLE-ABS-KEY("symptom manage*" OR "symptom self manage*" OR "management of symptom*" OR "symptom monitor*") |
| #2 | TITLE-ABS-KEY(“mobile application*” OR mobile* OR app OR apps OR “health application*” OR “mobile technolog*” OR “Cell Phone” OR “Cell Phone Use” OR phone* OR telephone* OR smartphone* OR “smart phone*” OR “smart device*” OR internet* OR “world wide web*” OR “web-based” OR “web based” OR “web-delivered” OR “internet delivered” OR “internet supported” OR “internet mediat*” OR “internet-based” OR “web page*” OR “web application*” OR “Virtual Medicine” OR telemedic* OR telehealth* OR “tele-medic*” OR “tele-health*” OR telemonitor* OR “tele-monitor*” OR ehealth* OR “e-health*” OR “electric health*” OR “m-health*” OR mhealth* OR “mobile health*” OR “e-therap*” OR etherap* OR “electronic pro” OR “electronic prom” OR epro OR eprom OR “e-pro” OR “e-prom” OR “electronic patient reported outcome*” OR “digital*” OR “intelligen*” OR “Remote Consultation” OR teleconsultation* OR “remote monitor*” OR “remotely monitor*” OR “Decision Making, Computer-Assisted” OR “Decision Making, Computer Assisted” OR “Computer-Assisted Decision Making” OR “Computer Assisted Decision Making” OR “Medical Decision Making, Computer-Assisted” OR “Medical Decision Making, Computer Assisted”) |
| #3 | TITLE-ABS-KEY("Neoplasms" OR Tumor* OR Neoplas* OR Cancer* OR Malignan* OR carcinoma*) |
| #4 | TITLE-ABS-KEY("Qualitative Research" OR qualitative research* OR qualitative stud* OR qualitative method* OR mixed research* OR mixed stud* OR mixed method* OR case stud* OR constant comparis* OR content analy* OR descriptive stud* OR phenomeno* OR grounded theor* OR focus group* OR ethnography OR narrative* OR interview* OR action research* OR participant observ* OR thematic analy* OR feminist OR naturalistic) |
| #5 | #1 AND #2 AND #3 AND #4 |
| **8. Search Strategy for ProQuest** | |
| #1 | abstract("symptom manage*" OR "symptom self manage*" OR "management of symptom*" OR "symptom monitor*") |
| #2 | abstract("Mobile Applications" OR “mobile application*” OR mobile* OR app OR apps OR “health application*” OR “mobile technolog*” OR "Cell Phone" OR “Cell Phone Use" OR phone* OR telephone* OR "Smartphone" OR smartphone* OR “smart phone*” OR “smart device*” OR “Internet” OR internet* OR “world wide web*” OR “web-based” OR “web based” OR “web-delivered” OR “internet delivered” OR “internet supported” OR “internet mediat*” OR “internet-based” OR “web page*” OR “web application*” OR "Telemedicine" OR "Virtual Medicine" OR telemedic* OR telehealth* OR “tele-medic*” OR “tele-health*” OR telemonitor* OR “tele-monitor*” OR ehealth* OR “e-health*” OR “electric health*” OR “m-health*” OR mhealth* OR “mobile health*” OR “e-therap*” OR etherap* OR “electronic pro” OR “electronic prom” OR epro OR eprom OR “e-pro” OR “e-prom” OR “electronic patient reported outcome*” OR “digital*” OR “intelligen*” OR “Remote Consultation” OR teleconsultation* OR “remote monitor*” OR “remotely monitor*” OR "Decision Making, Computer-Assisted" OR “Decision Making, Computer Assisted” OR “Computer-Assisted Decision Making” OR “Computer Assisted Decision Making” OR “Medical Decision Making, Computer-Assisted” OR “Medical Decision Making, Computer Assisted”) |
| #3 | abstract("Neoplasms" OR Tumor* OR Neoplas* OR Cancer* OR Malignan* OR carcinoma*) |
| #4 | abstract("Qualitative Research" OR qualitative research* OR qualitative stud* OR qualitative method* OR mixed research* OR mixed stud* OR mixed method* OR case stud* OR constant comparis* OR content analy* OR descriptive stud* OR phenomeno* OR grounded theor* OR focus group* OR ethnography OR narrative* OR interview* OR action research* OR participant observ* OR thematic analy* OR feminist OR naturalistic) |
| #5 | #1 AND #2 AND #3 AND #4 |
| **9. Search Strategy for CBM** | |
| #1 | "移动应用"[不加权:扩展] OR "app"[常用字段:智能] OR "apps"[常用字段:智能] OR "移动设备"[常用字段:智能] OR "智能设备"[常用字段:智能] OR "应用程序"[常用字段:智能] |
| #2 | "便携式电话"[不加权:扩展] OR "智能手机"[不加权:扩展] OR "移动电话"[常用字段:智能] OR "手机"[常用字段:智能] OR "智能手机"[常用字段:智能] |
| #3 | "因特网"[不加权:扩展] OR "互联网"[常用字段:智能] |
| #4 | "远程医学"[不加权:扩展] OR "远程会诊"[不加权:扩展] OR "在线医疗"[常用字段:智能] OR "远程医疗"[常用字段:智能] OR "移动医疗"[常用字段:智能] OR "电子医疗"[常用字段:智能] |
| #5 | "决策, 计算机辅助"[不加权:扩展] OR "计算机辅助"[常用字段:智能] OR "医学决策"[常用字段:智能] |
| #6 | "epro"[常用字段:智能] OR "eprom"[常用字段:智能] OR "电子化"[常用字段:智能] OR "智能化"[常用字段:智能] OR "数字化"[常用字段:智能] OR "患者报告结局信息测量系统"[常用字段:智能] OR "信息系统"[常用字段:智能] OR "ehealth"[常用字段:智能] OR "mhealth"[常用字段:智能] |
| #7 | #1 OR #2 OR #3 OR #4 OR #5 OR #6 |
| #8 | "症状管理"[常用字段:智能] OR "症状监测"[常用字段:智能] |
| #9 | "肿瘤"[不加权:扩展] OR "癌症"[常用字段:智能] OR "癌"[常用字段:智能] OR "瘤"[常用字段:智能] |
| #10 | "质性研究"[常用字段:智能] OR "扎根理论"[常用字段:智能] OR "焦点小组"[常用字段:智能] OR "参与观察"[常用字段:智能] OR "现象学"[常用字段:智能] OR "行为学"[常用字段:智能] OR "民族志"[常用字段:智能] OR "人种学"[常用字段:智能] OR "混合研究"[常用字段:智能] OR "定性研究"[常用字段:智能] OR "访谈"[常用字段:智能] OR "行动学"[常用字段:智能] |
| #11 | #7 AND #8 AND #9 AND #10 |
| **10. Search Strategy for CNKI** | |
| #1 | SU %= 移动应用 + app + apps + 移动设备 + 智能设备 + 应用程序 + 便携式电话 + 智能手机 + 移动电话 + 因特网 + 互联网 + 远程医学 + 远程会诊 + 在线医疗 + 远程医疗 + 移动医疗 + 电子医疗 + 计算机辅助 + epro + eprom + 电子化 + 智能化 + 数字化 + 患者报告结局信息测量系统 + 信息系统 + ehealth + mhealth |
| #2 | SU %= 症状管理 + 症状监测 |
| #3 | SU % = 肿瘤 + 癌症 + 癌 + 瘤 |
| #4 | SU % =质性研究 + 扎根理论 + 焦点小组 + 参与观察 + 现象学 + 行为学 + 民族志 + 人种学 + 混合研究 + 定性研究 + 访谈 + 行动学 |
| #5 | #1 AND #2 AND #3 AND #4 |
| **11. Search Strategy for Wan Fang** | |
| #1 | 主题:( 移动设备 OR 智能设备 OR 应用程序 OR 智能手机 OR 互联网 OR 移动医疗 OR 电子医疗 OR 计算机辅助 OR epro OR 数字化 OR 患者报告结局信息测量系统) |
| #2 | 主题:( 症状管理 OR 症状监测) |
| #3 | 主题:( 肿瘤 OR 癌症 OR 癌 OR 瘤) |
| #4 | 主题:(质性研究 OR 扎根理论 OR 焦点小组 OR 参与观察 OR 现象学 OR 行为学 OR 民族志 OR 人种学 OR 混合研究 OR 定性研究 OR 访谈 OR 行动学) |
| #5 | #1 AND #2 AND #3 AND #4 |
| **12. Search Strategy for VIP** | |
| #1 | 题名或关键词=移动应用 OR app OR apps OR 移动设备 OR 智能设备 OR 应用程序 OR 便携式电话 OR 智能手机 OR 移动电话 OR 因特网 OR 互联网 OR 远程医学 OR 远程会诊 OR 在线医疗 OR 远程医疗 OR 移动医疗 OR 电子医疗 OR 计算机辅助 OR epro OR eprom OR 电子化 OR 智能化 OR 数字化 OR 患者报告结局信息测量系统 OR 信息系统 OR ehealth OR mhealth |
| #2 | 题名或关键词=症状管理 OR 症状监测 |
| #3 | 题名或关键词=肿瘤 OR 癌症 OR 癌 OR 瘤 |
| #4 | 题名或关键词=质性研究 OR 扎根理论 OR 焦点小组 OR 参与观察 OR 现象学 OR 行为学 OR 民族志 OR 人种学 OR 混合研究 OR 定性研究 OR 访谈 OR 行动学 |
| #5 | #1 AND #2 AND #3 AND #4 |
