# Supplementary material for: Experiences of Patients With Cancer Using Electronic Symptom Management Systems: Qualitative Systematic Review and Meta-Synthesis
Source: J Med Internet Res. 2024 Oct 28;26:e59061. doi: 10.2196/59061 (PMC11555449; doi:10.2196/59061)
Supplement: Multimedia Appendix 4 [file jmir_v26i1e59061_app4.docx]

| **Extracted data** | **Definition** |
| --- | --- |
| **Study Characteristics** | |
| Author | The first author of the study. |
| Year of publication | The year in which the study was published. |
| Country/region of publication | The country/region where the study was conducted (where was the data collected). |
| Study design | What is the type of the study (mixed methods study or qualitative study, etc..)? |
| Aims | What is the aim of the study？ |
| Data collection | What is the data collection method for the study (semi-structured interview, focus-group discussion, etc..)? |
| Data analysis | What is the data analysis method used in the study (interpretative phenomenological analysis, thematic analysis, content analysis, etc..)? |
| **Participants Characteristics** | |
| Type of cancer | What is the caner type of participants in the study? |
| Type of treatment | What is the anti-cancer treatment of participants in the study? |
| Number of participants | What is the number of participants in the study? |
| Mean age | What is the mean age of the participants in the study? |
| Age range | What is the age range of participants in the study? |
| **Electronic symptom management systems (ESMSs) characteristics** | |
| Instruments for symptom measurement | What instruments are used for symptom assessment? |
| Function modules | What is the function of the ESMSs (e.g., assess symptoms, response to symptoms, generate a chart of symptom report history, provide advice on symptoms and self-care, etc..)? |
| Reporting timepoints | What is the timepoint/frequency of symptom assessment (daily, weekly, etc..)? |
| Duration of ESMSs use | How long was the ESMSs use by participants in the study (a week, a month, two months, etc..)? |
| **Outcomes** | |
| Qualitative findings | What are the qualitative findings of the study? |
| Quotations | What are the quotations of the findings in the study? |
