# Supplementary material for: Experiences of Patients With Cancer Using Electronic Symptom Management Systems: Qualitative Systematic Review and Meta-Synthesis
Source: J Med Internet Res. 2024 Oct 28;26:e59061. doi: 10.2196/59061 (PMC11555449; doi:10.2196/59061)
Supplement: Multimedia Appendix 5 [file jmir_v26i1e59061_app5.docx]

| **Reference** | **Q1** | **Q2** | **Q3** | **Q4** | **Q5** | **Q6** | **Q7** | **Q8** | **Q9** | **Q10** | **score** |
| --- | --- | --- | --- | --- | --- | --- | --- | --- | --- | --- | --- |
| Darley A et al [20], 2023 | Y | Y | Y | Y | Y | N | Y | Y | Y | Y | 9 |
| Maguire R et al [21], 2020 | Y | Y | Y | Y | Y | N | Y | Y | Y | Y | 9 |
| Crafoord MT et al [23], 2020 | Y | Y | Y | Y | Y | N | Y | Y | Y | Y | 9 |
| Lapen K et al [24], 2021 | Y | Y | Y | Y | Y | C | Y | Y | Y | Y | 9.5 |
| Maguire R et al [25], 2015 | Y | Y | Y | Y | Y | N | Y | Y | Y | Y | 9 |
| Langius-Eklöf A et al [30],2017 | Y | Y | Y | Y | Y | Y | Y | Y | Y | Y | 10 |
| Sundberg K et al [31], 2015 | Y | Y | Y | Y | Y | N | Y | Y | Y | Y | 9 |
| McCann L et al [43], 2009 | Y | Y | Y | Y | C | N | Y | Y | Y | Y | 8.5 |
| Richards HS et al [44], 2021 | Y | Y | Y | Y | Y | N | C | Y | Y | Y | 8.5 |
| Richards HS et al [45], 2020 | Y | Y | Y | Y | Y | N | Y | Y | Y | Y | 9 |
| Lattie EG et al [46], 2020 | Y | Y | Y | Y | Y | N | Y | Y | Y | Y | 9 |
| Gomaa S et al [47], 2023 | Y | Y | Y | Y | Y | N | Y | Y | Y | Y | 9 |
| McCready TM et al [48], 2023 | Y | Y | Y | Y | Y | N | Y | Y | Y | Y | 9 |
| Erickson JM et al [49], 2019 | Y | Y | Y | Y | Y | N | Y | Y | Y | Y | 9 |
| Gustavell T et al [50], 2019 | Y | Y | Y | Y | Y | N | Y | Y | Y | Y | 9 |
| Whitehead L et al [51],2020 | Y | Y | Y | Y | Y | N | Y | Y | Y | Y | 9 |
| Pereira-Salgado A et al [52], 2017 | Y | Y | Y | Y | Y | N | Y | Y | Y | Y | 9 |
| Chan CW et al [53], 2011 | Y | Y | Y | Y | Y | N | Y | Y | Y | Y | 9 |
| Tang FWK et al [54], 2018 | Y | Y | Y | Y | Y | N | Y | Y | Y | Y | 9 |
| Moradian S et al [55], 2018 | Y | Y | Y | Y | Y | N | Y | Y | Y | Y | 9 |
| Mirkovic J et al [56], 2014 | Y | Y | Y | Y | Y | N | C | Y | Y | Y | 8.5 |

Q1 Was there a clear statement of the aims of the research?

Q2 Is a qualitative methodology appropriate?

Q3 Was the research design appropriate to address the aims of the research?

Q4 Was the recruitment strategy appropriate to the aims of the research?

Q5 Was the data collected in a way that addressed the research issue?

Q6 Has the relationship between researcher and participants been adequately considered?

Q7 Have ethical issues been taken into consideration?

Q8 Was the data analysis sufficiently rigorous?

Q9 Is there a clear statement of findings?

Q10 How valuable is the research?

Y=YES C=Can’t Tell N=NO

Reference: Critical Appraisal Skills Programme (2018). CASP Qualitative Checklist. [online] Available at: https://casp-uk.net/checklists/casp-qualitative-studies-checklist-fillable.pdf. Accessed: 2023-05-30.
