# Supplementary material for: Experiences of Patients With Cancer Using Electronic Symptom Management Systems: Qualitative Systematic Review and Meta-Synthesis
Source: J Med Internet Res. 2024 Oct 28;26:e59061. doi: 10.2196/59061 (PMC11555449; doi:10.2196/59061)
Supplement: Multimedia Appendix 6 [file jmir_v26i1e59061_app6.docx]

| **author (year)** | **Country & region** | **design** | **aims** | **type of cancer & treatment** | **sample (median age)** | **instruments for symptom measurement** | **function modules** | **reporting timepoints & study duration** | **data collection** | **data analysis** |
| --- | --- | --- | --- | --- | --- | --- | --- | --- | --- | --- |
| Darley A et al [20], 2023 | Ireland | a longitudinal, multi-perspective interpretative phenomenological analytical qualitative design | Explore the lived experiences of receiving chemotherapy using digital health symptom management technology | Colorectal cancer | males=2, females=1; age range 70~78 | Memorial Symptom Assessment Scale; Functional Assessment of Cancer Therapy-General; State-Trait Anxiety Inventory-Revised | 1. Assessment of symptoms based on self-report.  2. Medical staff respond to symptoms based on severity of the systems.  3. Provide advice on symptoms and self-care | Daily; 6 cycles | One-to-one in-dept interviews | Interpretative phenomenological analysis |
| Maguire R et al [21], 2020 | England and Scotland. | convergent mixed method study | Adapt ASyMS for people with MPM (ASyMSmeso) and determine the feasibility and acceptability of integrating ASyMSmeso into oncology care delivery. | Malignant pleural mesothelioma (MPM) | males=6, females=2; mean age 71 | Self - designed questionnaire-Daily Symptom Questionnaire including breathlessness, pain, cough, sweating, fatigue, appetite, issues with indwelling pleural catheters, and constipation | 1. Assessment of symptoms based on self-report.  2. Detect risk symptoms and automatic alarms.  3. Doctors respond to symptoms based on alerts.  4. Provide self-care advice.  5. Generate a chart of symptom report history | Daily and at any time they feel unwell; 3months (data were collected at baseline, 6 weeks and the end of the study), then submitted and ethical amendment asking to reduce the period to 2 months (data were collected at baseline and the end of study only) | Semi-structured interview and focus groups | Thematic analysis as advocated by Braun and Clark |
| Crafoord MT et al [23], 2020 | Sweden | mixed methods study | Describe engagement with the Interaktor app during treatment. | Breast cancer or prostate cancer; neoadjuvant chemotherapy and radiotherapy, respectively | females with breast cancer=73, males with prostate cancer=58 | Memorial Symptom Assessment Scale | 1. Assessment of symptoms based on self-report.  2. Detect risk symptoms and automatic alarms.  3. Nurses contact patients based on symptoms alerts.  4. Provide self-care advice.  5. Generate a chart of symptom report history | Daily on weekdays; breast cancer: 18weeks (on their first day of neoadjuvant chemotherapy and continue until 2 weeks after treatment had ended), prostate cancer: 9weeks(on their first day of radiotherapy and continued until 3 weeks) | A semi-structured telephone interview | Conventional content analysis |
| Lapen K et al [24], 2021 | America | mixed method study | Identify factors influencing ePRO completion in the setting of breast radiation, understand patient and clinician preferences for a symptom response system | Breast cancer; radiation to the breast or chest wall | n=15 | Self - designed questionnaire-based on the Patient-Reported Outcomes Common Teminology Criteria for Adverse Events (PRO-CTCAE) including skin changes, pain in the radiated area, swelling, tenderness, fatigue, and pain with swallowing; also included questions about anxiety | 1. Assessment of symptoms based on self-report.  2. Detect risk symptoms and automatic alarms based on symptom severity.  3. The care team response to symptoms based on alerts. | Weekly; during treatment and for 8 weeks following the completion of treatment. | Semi-structured, qualitative interviews | A matrix analysis |
| Maguire R et al [25], 2015 | the UK | mixed methods study | Develop and explore the feasibility and acceptability of the ASyMS | lung cancer, thoracic radiotherapy | males=5, females=11; mean age 63.6 (range, 42~85) | Edmonton Symptom Assessment Scale; The State-Trait Anxiety Inventory Form Y; Functional Assessment of Cancer Therapy-Lung Cancer; | 1. Assessment of symptoms based on self-report.  2. Detect risk symptoms and automatic alarms.  3. Provide evidence-based self-care advice.  4. health care provider contact patients based on alarms. | Daily; for the duration of their radiotherapy treatment and for one month post-treatment | Semi-structured interviews | Thematic content analysis |
| Langius-Eklöf A et al [30],2017 | Sweden | mixed method study | Investigate user behavior, adherence to reporting, and experiences of using Interaktor | locally advanced prostate cancer; planned for radiotherapy | males=53 | Self - designed questionnaire including 14 identified and tested symptom questions regarding bladder (urinary urgency, difficulties in urinating, urinary leakage, and hematuria) and bowel (diarrhea, stool leakage, obstipation, and blood in stool) function, fatigue, pain, worry, depression, sleep, and flushing and other symptoms or concerns to report | 1. Assessment of symptoms based on self-report.  2. Detect risk symptoms and automatic alarms.  3. Provide self-care advice.  4. Generate a chart of symptom report history. | Daily (or more often if they wished); during office hours on weekdays throughout the radiotherapy period and for 3 weeks after | Telephone and face-to-face interviews | Summative content analysis |
| Sundberg K et al [31], 2015 | Sweden | mixed methods study | Test the feasibility and acceptability of an interactive ICT-platform for smartphone use which collect and manage patient reported symptoms | Prostate cancer; radiotherapy | males=9 | Self - designed questionnaire including urinary urgency, urinary pain, difficulties urinating, hematuria, urinary leakage, diarrhea, obstipation, stool leakage, blood in stool, fatigue, insomnia, depressed, worry | 1. Assessment of symptoms based on self-report.  2. Detect risk symptoms and automatic alarms.  3. Nurses contact patients based on symptoms alerts.  4. Provide self-care advice.  5. Generate a chart of symptom report history | Daily; two weeks | A focus-group discussion or individual interviews | Qualitative content analysis |
| McCann L et al [43], 2009 | the UK | mixed method study | Evaluate the impact of a mobile phone-based, remote monitoring ASyMS© | Lung, breast or colorectal cancer; outpatient chemotherapy | n=12 | Self - designed questionnaire including six symptoms (fatigue, nausea and vomiting, oral problems, diarrhoea and breathlessness) | 1. Assessment of symptoms based on self-report.  2. Detect risk symptoms and automatic alarms.  3. Provide self-care advice.  4. Health professionals contact patients based on alarms. | Twice a day; On days 1–14 following their first four cycles of chemotherapy (in the morning, evening and at any time they felt unwell) | Semi-structured interviews | Thematic content analysis |
| Richards HS et al [44], 2021 | The UK | qualitative study | Understand participants’ experiences and perceptions of using the ePRO system after hospital discharge following UGI cancer-related surgery to support their symptom management. | Upper gastrointestinal cancer; undergone related surgery, were ready for hospital discharge to their home | males=11, females=5; mean age 63 (range, 43~73) | European Organization for Research and Treatment of cancer (EORTC) questionnaires: C30 (core cancer quality of life module), OG25 (oesophago-gastric cancer module), OES18 (oesophageal cancer module), LMC21 (colorectal liver metastases module), HCC18 (hepatocellular carcinoma module) and 5 additional items(e.g. wound problems) | 1. Assessment of symptoms based on self-report.  2. Detect risk symptoms and automatic alarms.  3. Provide self-care advice.  4. Generate recommendations to contact medical practitioners based on alerts.  5. Generate a chart of symptom report history | Twice in the first week and weekly for 8 weeks post discharge | Weekly telephone interview: Semi-structured telephone interviews; End-of-study interview: a face-to-face interview | Thematic analysis was conducted in accordance with Braun & Clarke’s guidelines |
| Richards HS et al [45], 2020 | England | mixed methods prospective pilot study | Examine participants’ perspectives on the usefulness of the ePRO system and feedback from the perspective of examining the feasibility and usability of the system. | Patients had undergone cancer-related UGI surgery | males=19, females=10; mean age 64 (range, 43~81) | European Organization for Research and Treatment of cancer (EORTC) questionnaires: C30 (core cancer quality of life module), OG25 (oesophago-gastric cancer module), OES18 (oesophageal cancer module), LMC21 (colorectal liver metastases module), HCC18 (hepatocellular carcinoma module) and 5 additional items(e.g. wound problems) | 1. Assessment of symptoms based on self-report.  2. Detect risk symptoms and automatic alarms.  3. Provide self-care advice.  4. Generate recommendations to contact medical practitioners based on alerts.  5. Generate a chart of symptom report history | Twice in the first week post-discharge and weekly (and at additional timepoints if they wished) for 8 weeks thereafter | Weekly telephone interview: Semi-structured telephone interviews; End-of-study interview: a face-to-face interview | Thematic analysis |
| Lattie EG et al [46], 2020 | America | mixed method approach | Evaluate the usability of this new health information technology tool in preparation for its implementation | Cancer | males=5, females=5; mean age 55.3 (range, 39~74) | Self - designed questionnaire including information on common cancer-related symptoms (eg, pain, fatigue, nausea, physical functioning, insomnia, anxiety, depression, constipation) | 1. Assessment of symptoms based on self-report.  2. Provide self-care advice.  3. Contact medical staff on the system.  4. Create favorites. | Following prototype testing, all participants participated in a brief (approximately 15 minutes) interview that addressed participant impressions of My NM Care Corner | Interview | Conceptual content analytic approach |
| Gomma S et al [47], 2023 | America | mixed method approach | Develop and implement a text messaging–based system, Real-time Chemotherapy-Associated Side Effects Monitoring Supportive System (RT-CAMSS) | Patients with gastrointestinal receiving chemotherapy | n=9 | 4 elaborated symptoms categories (constitutional and general, cardiovascular and respiratory, gastrointestinal, neurological) | 1. Proactive Interactive Text Messages.  2. Chatbot Symptom Monitoring.  3. Immediate Self-Care Feedback | Weekly; 2 months | Semi-structured phone interviews | / |
| McCready TM et al [48], 2023 | America | qualitative study | Describe the patient experience using the symptom monitoring tool, the Recovery Tracker | Cancer patients; surgery | males=17, females=26; median age 56 (range, 38~75) | 11 items adapted from the National Cancer Institute (NCI)’s Patient-Reported Outcomes version of the Common Terminology for Adverse Events (PRO-CTCAE), three additional surgical symptom questions | 1. Assessment of symptoms.  2. Detect risk symptoms and automatic alarms.  3. reflect on the alert. | Daily; 10days | Qualitative phone interview | Content analyses |
| Erickson JM et al [49], 2019 | the US | longitudinal mixed methods study | Examine the effects of a heuristic symptom assessment tool, the Computerized Symptom Capture Assessment Tool (C-SCAT), on AYAs’ self-efficacy for symptom management, their self-regulation abilities related to symptoms, and negotiated collaboration, as operationalized | Cancer; myelosuppressive chemotherapy, completion of at least 1 month of chemotherapy with at least two additional treatments anticipated | males and females | Not mentioned | 1. Assessment of symptoms based on self-report.  2. Identify temporal and causal relationships between symptoms and symptom clusters.  3. Name symptom clusters and identify priority symptoms and a priority symptom cluster. | At the next two scheduled visits for chemotherapy (visits 1 and 2) and before meeting with their provider. | Semi-structured interview | Qualitative content analysis |
| Gustavell T et al [50], 2019 | Sweden | qualitative study | Develop and test a version of the Interaktor app adapted for patients who have undergone pancreaticoduodenectomy | Patients who have undergone pancreaticoduodenectomy | males=3, females=3; mean age 65 (range, 57~74) | Self - designed questionnaire including 12 common symptoms (fever, eating difficulties, nausea, vomiting, loose stool, constipation, pain, dizziness, fatigue, anxiety and worry, and problems with daily activities at home and outside the home) | 1. Assessment of symptoms based on self-report.  2. Detect risk symptoms and automatic alarms based on symptom frequency and disturbance.  3. Nurses contact patients based on alarms.  4. Provide evidence-based self-care advice.  5. Generate a chart of symptom report history. | Daily; for 4 weeks starting the first day after discharge from the surgical or rehabilitation clinic. | Semi-structured interview | Qualitative content analysis |
| Whitehead L et al [51],2020 | Australia | mixed methods study | Develop and implement a SAM web app | Breast cancer or colorectal cancer; completed surgery, and were scheduled to receive adjuvant chemotherapy, receiving a minimum of 3 cycles of chemotherapy on an outpatient basis | males=5, females=19 | Functional Assessment of Cancer Therapy-Breast; Functional Assessment of Cancer Therapy-Colorectal; the Rotterdam Symptom Checklist; the Hospital Anxiety and Depression Scale | 1. Assessment of symptoms based on self-report.  2. Provide immediate feedback on self-care actions.  3. Generate a chart of symptom report history.  4. Provide a repository of evidenced based information on key symptoms | Daily or anytime a participant wanted to assess their symptom; phase 2, pilot involving one cycle of chemotherapy, and phase 3, intervention over 3 cycles of chemotherapy. | Simi-structured phone interview | Content and thematic analysis |
| Pereira-Salgado A et al [52], 2017 | Australia | mixed method study | Develop and pilot-test the clinical feasibility and acceptability of a mobile health system (REMIND) to increase oral drug adherence and patient symptom self-management among people with CML (chronic phase). | Chronic-phase CML with no signs of progression; >3 months of continuous treatment with imatinib (Glivec) with no evidence of drug resistance | males=6, females=3; median age 54 (range, 35~72) | Not mentioned | 1. Assessment of symptoms based on self-report.  2. Generate daily medication reminders.  3. Provide evidence-based self-care advice. | Over 10 weeks; daily respond to a text reminder message for each dose based on his or her individual regimen and weekly complete an online symptom survey. | Semi-structured telephone interview | Content analysis |
| Chan CW et al [53], 2011 | China | mixed method study | 1. Assess the acceptability and feasibility of using ESRA-C 2. Identify factors that affect the acceptability and feasibility of ESRA-C | Cancer; ambulatory, currently experiencing cancer symptoms | n=27 | European organization for research and treatment of Cancer; Symptom Distress Scale (SDS); Pain-Intensity Numerical Scale; Patient Health Questionnaire-depression module (PHQ-9) | 1. Assessment of symptoms based on self-report. | Use only once in a public area of the hospital and then be interviewed | Semi-structured qualitative interviews | Content analysis |
| Tang FWK et al [54], 2018 | China | mixed method study | Assess the feasibility and acceptability of tablet-assisted self‐reported symptom assessment | Lung cancer | males=6; females=4; mean age 66.7 (range, 53~89) | The Modified Borg scale; the Chinese version of the Brief Fatigue Inventory; the Chinese version of the Brief Pain Inventory; the Chinese version of the State-Trait Anxiety Inventory | 1. Assessment of symptoms based on self-report.  2. Provide data export.  3. Provide symptom management advice. | Participants self-reported symptom assessment while they waited for their consultations at the out-patient clinic | Semi‐structured interviews | Content analysis |
| Moradian S et al [55], 2018 | Canada | mixed methods study | Evaluate the usability of the Advanced Symptom Management System (ASyMS), explore users’ performance and satisfaction with the system interface and their perspectives and experience with the system and the content of ASyMS | Colorectal cancer or lymphoma; chemotherapy | males=7, females=3; mean age 68 (range, 18~78) | Not mentioned | 1. Assessment of symptoms based on self-report.  2. Detect risk symptoms and automatic alarms.  3. Provide self-care advice.  4. Clinicians contact patients by telephone based on alarms. | Usability testing sessions | Face-to-face semi-structured qualitative interviews | Thematic analysis |
| Mirkovic J et al [56], 2014 | Norway | mixed method study | Evaluate the usability of the Connect Mobile app. The mobile app enables mobile access to the Connect system, an online system that supports cancer patients in managing health-related issues. | Cancer survivors | males=3, females=4; mean age 61 (range, 49~75) | Not mentioned | 1. Assessment of symptoms based on self-report.  2. Generate a chart of track of problems that are bothering patients.  3. Provide self-care advice.  4. Communicate with medical staff.  5. Exchange information and experiences with other patients.  6. Create free text for private health-related information. | Usability testing sessions in a quiet room at a community center with a moderator (JM). | Semi-structured interviews | Thematic analysis |
