# Supplementary material for: Experiences of Patients With Cancer Using Electronic Symptom Management Systems: Qualitative Systematic Review and Meta-Synthesis
Source: J Med Internet Res. 2024 Oct 28;26:e59061. doi: 10.2196/59061 (PMC11555449; doi:10.2196/59061)
Supplement: Multimedia Appendix 7 [file jmir_v26i1e59061_app7.docx]

| **Category 1: Perceptions and attitudes towards ESMSs** | |
| --- | --- |
| **Subcategory1: Ease of use** | |
| **Easy to use and navigate ESMSs** | [25] “I had no hassle with it at all, and as I say having, you know I'm no(t) great on computers and things but I have some knowledge so I was able to do it”.  [46] “Well it’s not, it’s not hard to navigate at this point, per se. At this point I mean, it’s pretty logical.” |
| **able to use ESMSs smoothly if they were trained or use them a few times.** | [50] “Everything has worked fine, except in the beginning. I don’t know if I did something wrong, but I didn’t manage to log in at first. My own phone is old, and I’m not used to these new ones. But then one of the researchers came and showed me how to do it, and after that it has worked fine.”  [55] “If people use this a few times they will be able to (use and) navigate it easily. "  [56] “I think I will be able to use [the app], but it will take time. These tasks…When it is new then there are a lot [of new things]. However, I did this in the Connect [Web app]. So I can find what I want now [in the system].” |
| **Easy to understand the terms of symptom** | [20] "It’s in everyone’s language, we’ll say, it’s not doctor language or anything. It’s simple." |
| **more convenient compared with pen-and -paper formats and face-to-face assessments** | [50] “If you clicked on the wrong answer, you could easily go back and correct yourself.”  [54] "The content is very clear. I followed each (assessment) item one by one. On the other hand, if you read to me, I may not be able to follow you." |
| **Gain a better understanding and mastery of self-management information** | [54] "… use the tablet to show video clips. This way clearly shows the skills. Patients may find it easier to understand… Compared with using text only, it's easier for patients… it's like learning tai chi, you can't read the text only. You need someone to show you and then you follow. That makes things easier." |
| **Subcategory 2: Support symptom monitoring and management** | |
| **Enabled and prompted them to describe symptoms accurately** | [49] "...makes it easier to find the words that I’m looking for instead of sitting here going uh how do I explain this to her?”  [50] “There were several alternatives to choose from, which were really good because I could exactly pinpoint how I felt when I sent in my report.” |
| **helped them recall symptoms in general** | [20] “For me, I think so yeah, because you get the symptoms to look for, the pins and needles and things like that, but there’s other stuff in there on the phone that’s listed, and they do tell you, but you forget. Advise you if you’ve had diarrhoea, constipation, any problem with your feet, which I’ve had, that sort of thing. "  [49] "Well, maybe if you couldn’t remember what was wrong, it would be right there or something that you forgot. I only come once a month so sometimes it helps to remind me what is going on.” |
| **Were good at telling them what’s normal or not** | [45] “The system’s good at telling you what’s normal, or not even normal, but that other people have this [experience of symptoms].” |
| **Symptoms were being tracked by someone** | [20] “If there’s a problem, someone will ring you.”  [46] “I like the idea of the dashboard a lot. I mean it’s nice to know that when I provide information it’s not just going onto some file somewhere and no one’s really reading it.”  [48] “…you know you’re not alone. It’s like ok, is this all right that I’m feeling this way? …And I knew I was being monitored, so if there was something that was off…they would’ve contacted me and said, ‘Listen this is not ok. ’ Or I would’ve gotten a prompt on the computer…that said, ‘You might want to call your doctor’… The fact that you guys were monitoring my recovery made me feel more secure. ”  [52] “It was good...if someone (nurse) noticed a change in symptoms I might not have picked up on it, and they were able to discuss it with me and find out why it might have happened.” |
| **Graphs of symptoms in ESMSs enabled them to be aware of their symptoms and track symptoms over time** | [44] “You don’t see them in isolation as one week, you see [graphs of symptom reports] as a pattern of progress and that’s the way to look at them…when I look along the row from where I was to begin with to now where most [symptoms] are at zero I’m pleased.”  [51] “Just the fact that you can go back and just having a little bit of a good look at the history you know that sometimes you can’t quite remember sometimes. When you had a bad day or something you can go back and go you can see your peaks and troughs—these things that you’ve selected. I’ve gone back and had a look and see how it goes up and down. [referring to graphs] cos you forget sometimes when your good days are and your bad days." |
| **reinforce medical guidance and remind them to follow information** | [44] “I must have read it [patient information leaflet] in the first week or so and I’d forgotten all about it. Yeah…[I’ll] try to follow that [ePRO system] advice… It’s reminded me and [I’ve] thought I have got some medication for that, I should be taking it.”  [45] “[The same advice] had been mentioned earlier on in the process, I think when I had my initial consultation…and it just reminded me of all those things…And I found them very useful, although they weren’t adding anything new, again they were reassuring.” |
| **The self-care information was specific, appropriate and achievable** | [44] “At the end it all sort of comes together and it gives you advice…It works well…and aimed at me specifically.”  [47] “It told me a lot about my loss of appetite and this was really helpful, it helped in how to deal with issues and side effects.” |
| **Subcategory 3: Symptom reporting becomes a part of their daily routines** | |
| **Became embedded in participants’ daily routines** | [20] “Well, it’s em, it’s normal if you like. I get up, I have my breakfast, I have a wash and then I come out and I do the questionnaire straight away. That’s my rote in the morning. Get up, have a wash, have my breakfast, then sit down and do the questions. It’s no imposition on my time really. I do it very quickly.”  [43] “It was very easy, it was very simple to do and eh, it didn’t really encroach on lifestyle or anything like that at all, just had to remember to do it [laughs], set the ‘pinger’ on the cooker.” |
| **Subcategory 4: Negative Perspectives** | |
| **Questioned the clinical relevance** | [24] “I didn’t see the worth... Towards the end of treatment I told somebody, ‘don’t ask me, I don’t want to participate in any more studies” |
| **Concern that their reports would be ignored** | [53] “Useful or not just depends on how seriously the medical practitioners took the report. If they just put it aside, it will be useless and a waste of my time to fill in the questionnaire.” |
| **Disappointed at not being contacted and doubted the trustworthiness** | [30] “I was disappointed when no one called...it seems questionable whether the system can be trusted.” |
| **Preferred face-to-face communication** | [54] “When doctors or nurses directly ask me about my disease, they seem to be more reliable. I feel more secure in conveying my concerns to a doctor… It seems to be more dependable to talk to a person.”  [54] “The assessment is better done by doctors. They know about my disease and are more professional.” |
| **Concerned about the additional workload and pressure for doctors** | [53] “I am afraid it is too harsh for doctors. It is because they have to spend much time to track the history of each individual, a single simple question can cost them a lot of time to figure out the answer.” |
| **The self-care information in ESMSs was similar to what they got** | [25] “Aye, I'll be honest about that, I scanned over it [self-care] because I was getting, I, I was getting the treatment for it, do you know I was doing, what I should have been doing kind of thing it wis'nae [was not] anything that was new to me.” |
| **Category 2: The value of ESMSs** | |
| **Subcategory 1: Increasing motivation for symptom monitoring and management** | |
| **Understood of the causes and effects of symptoms** | [30] “When I answered the questions, I thought a lot about how I was feeling...It gave me perspective on my illness...I was feeling pretty good after all...”  [49] “I liked noting which ones were a priority in the groups because it was nice to be able to say this is the worst of it.”  [50] “The app gave me a daily routine to think about what I can manage. If I’m in pain, I think about my response, and I think OK, maybe I walked too fast yesterday.”  [54] “I think it's different from the usual practice. After using this device (tablet PC), I know more about myself..” [54] “This approach helps me to be aware of my own symptoms and condition. It's really helpful to me.” |
| **Stimulated for thinking about how to manage and/or prevent symptoms** | [49] "By knowing what causes what I’m able to try to help prevent the first symptom that causes everything else. So if I can stop that then hopefully I can stop the rest. "  [51] “Yes there would have been times where I went Ah don’t worry I’ll feel okay I just won’t bother with my temperature. Yeah. And I think that was a big eye opener…So it made me go stop, rest and recheck myself." |
| **Helped improve adherence** | [52] "...it definitely helped reinforce habit when I was at the start of getting on, getting this medication into routine.”  [52] "I’ve had overseas visitors for the last 2 weeks so I’m out of my usual routine…I’m sure I would have missed some of my drug taking…had I not had the reminders." |
| **Subcategory 2: Enhancing connections and effective communication with others** | |
| **Connection with healthcare providers was not interrupted** | [44] “So [ePRO system] makes you feel not cut off…Because otherwise I think the issue can be you get all this attention in hospital, and then suddenly that’s it – you’re off on your own.”  [50] “I have used the app as if I was having a conversation with a nurse.” |
| **Provided quick access to healthcare providers** | [25] “Well as far as I am concerned yes, because it was very helpful because I had this bad cough and one or two alerts came up and the nursing staff at the other end were immediately onto it... the fact that we were in contact with the hospital very much quicker than we would be if we’d waited and maybe even phoned.”  [31] “I was happy that the nurse called me. I did not have to go through issues of being connected or waiting in a telephone queue.”  [43] “It was fine. It seemed to be worthwhile. Seemed to be doing the kind of thing I would have expected it to try and do. As I say some people at the other end reacted when it flagged up a possible problem so I was quite impressed.” |
| **Increase chances of receiving further consultations and treatment** | [20] “It’s good, yeah, because you can talk to them and they can tell you like what to do. If it’s the sickness they tell you about the different tablets and to try and do such and such in between and that yeah, you know? "  [44] “I spoke to the [cancer nurse specialist] and she put me in touch with the dietitian. But that all followed on from my completing a questionnaire, so it prompted me to do it I think.” |
| **Helped prepare for consultations before their visits** | [49] "I think it was good because it made me kinda prepare—kinda run down what I am going through the past 24 hours."  [49]"…it reminds me to ask about these things or talk about these things." |
| **Saved time during medical consultations** | [53] “When you use the computer report, it will be clearer. It means the doctors do not need to listen to you for so long time. It is very convenient. Because the doctor won’t allow you to talk so much, now he just clicks the computer and knows. It saves a lot of time. It can help the patient, as well as the doctor.” |
| **Communicated with healthcare providers effectively** | [49] "It makes it easier to talk to them because they have it all in front of them and then they can go category to category. " |
| **Helped them share their feelings and symptoms with family and friends** | [30] "I used the graphs to show my family and friends that I actually felt good during the treatment. " |
| **Helped communication and cooperation with other patients** | [56] "It is not just the fact that health care personnel should be available and give you answer in 24 or 48 hours. But we should be help to each other… I think the overall goal of the system and its functionalities is the meeting point for cancer patients… So we can share experiences about our anxiety, treatment, diagnose… And they [the patients] can be of help to each other. " |
| **Subcategory 3: Gaining Positive psychological experiences** | |
| **Felt reassured** | [21] “it was reassuring to know that you’re feeling stable and not having symptoms.”  [23] "I felt safe reporting every day.... It was excellent…. I noticed, before logging off, that I would be called...A huge security… Having the app and a continuous access to help has made me feel better."  [51] "The information was you know that it sort of reassured me that things were going probably as they should do." |
| **Overcame their feelings of uncertainty and concern about symptoms** | [25] "I was very pleased because once you’re away from the hospital and you needed contact with them you’re out on a limb sort of thing… and you do tend to think “oh well perhaps this isn’t anything” and at least when you’re in contact with them (via ASyMS) they can, they know whether it is anything that’s necessary or not. So yes I quite agree with it, I’m glad that they were (there).”  [45] “I mean you get monitored so much in the hospital…And then you come out and there’s nothing at all. It’s like a sudden drop off a cliff really. And your first week and that you go well, am I OK? You know they’ve been checking for all this time.” |
| **Alleviated anxiety and nervousness** | [20] "I would imagine that I would be very stressed out. I’d imagine that I would be thinking all kinds of dire thoughts. I would imagine that I would be, to a very certain extent, left on my own. I certainly think that this is a very positive step in putting my mind at rest for the reasons I set out in that any query I have or question I have, there’s somebody there straight away. It’s back-up reassurance and certainly if I didn’t have this option or facility opened to me, I would not be as relaxed or as, or as, or as happy as I am, you know? " |
| **Felt they were being listened to and care for** | [21] "I think what was good was the fact that you always felt that there was somebody else at the other end of the line who was listening to what you were saying. "  [52] "...the good part...was the personal touch...it’s just that really nice reminder that there’s someone out there that’s half looking out for you. " |
| **Subcategory 4: Potential benefit for health care** | |
| **Reduce frequency in contacting hospitals** | [43] "Yes oh I definitely think that this should be something that’s taken on board as a backup and I’m sure it would possibly alleviate I wouldn’t say pressure on hospital staff but it would save people having to phone up the hospital whenever they do have any symptoms or any queries or worries . . . " |
| **Alleviated health care burdens** | [30] "It almost feels like having health care staff in one’s home. I think there may be some kind of...perhaps less burden on the health care. " |
| **Category 3: Barriers, requirements and suggestions for ESMSs** | |
| **Subcategory 1: Barriers to using ESMSs** | |
| **Older or with lower educational level** | [24] “It is harder for older women who don’t do all of the electronic stuff.”  [53] “I really don’t know how to type in Chinese. Also I am not good at English. For example, I want to report the symptoms which did not include in the survey, but I don’t know how to type.”  [54] “Those highly educated usually find it easier to accept new technology. Those less educated would encounter more difficulties.” |
| **Health conditions** | [51] "Well there were links and information that you could actually access direct from there. Sometimes I was just too tired to follow it through. But when I was able I appreciated the fact that the information was there. So I’ve actually kept the stuff in mind to take action. I’ve actually printed some of this stuff out so I know what to do if I get a um, what to watch out for."  [53] “At this point of time, I am fine to answer these questions, but I am not sure whether I will be physically fit to complete the assessment properly later since my disease is relatively aggressive. I have to undergo chemotherapy every week, so I might be physically or psychologically unwell to participate in the assessment.” |
| **Instability of ESMSs and networks** | [52] "There were maybe three times that the computer system either didn’t send a text or it was an hour or 2 late... " |
| **Subcategory 2: Requirements and suggestions for ESMSs** | |
| **Improve the simplicity and accuracy of the symptom assessment items** | [24] “Skin color, sensation, itchy, dry, skin breakdown. They are all kind of similar”  [30] "Relevant questions, but might be a little blunt; hard to know what is meaningful to report, hard to put the level of how to respond to such as “not at all” or “a little” distress in the beginning."  [53] “It has too many repetitions. For example, there are three questions related to vomiting.” |
| **Report additional symptoms** | [24] "I would have liked to see something that explores how the patient feels about the severity of the skin discoloration."  [43] "But there wis things I thought – noo, that’s how I feel and that’s what I’ve got but they’re no asking that, so I could’nae put it doon, do you know what I mean." |
| **Frequency of symptom assessment** | [43] "I think it’s a good idea to–to do it twice–twice a day, because with chemotherapy you can feel fine in the morning, not feel so good later on, or vice versa, you know? So I think, you know, it’s important to see what times of the day, you know, you’re feeling at your best, or your worst, or whatever."  [53] “I think it is adequate to take part in the assessment every six months. It is meaningless to participate in such assessment so frequently because the symptoms are more or less the same. it is annoying and troublesome if compulsory assessment participation is required every time prior to consultation with doctors.” |
| **An alert to remind logging in and record symptoms** | [43] "Maybe we should, have put a wee alarm on it to say, or it come up ‘you have not completed the questionnaire today’ or something like that."  [51] "Sometimes it was because I was feeling really rough and other times it was that I basically forgot. I should have done it…and I suppose if I hadn’t done it one day I thought I might have thought ‘did I do it’? I couldn’t remember whether I’d done it as well sometimes. I suppose that sometimes I’d go in and I’d do it—put my symptoms in and then I don’t think it logged it because it kinda logged me out and I had to log back in and then I’d repeat what I’ve just done." |
| **Entered data retrospectively and re-edit previously entered data** | [51] “The thing that I actually found the most frustrating to be honest, was the fact that and especially initially um you couldn’t go back. Like the first day I logged in and I couldn’t actually go back to like the couple of days before that and put in, like I’d noted down what my symptom were but I couldn’t go back because of it. Like you couldn’t do it for a specific date. I think would’ve been helpful for you guys and for me."  [51] “But again—also being able to edit it because sometimes I accidentally pressed like it went to 10 because I scrolled up and I accidentally pressed it not realizing yet because it would look like I’ve hit a 10 but I can’t go back and edit it." |
| **Desire to decide when or whether to be contacted, or to contact healthcare providers** | [30] "It took me about a week to fine tune the level at which to report symptoms. At the start the nurses called me pretty often, but then I learned how to report the symptoms so as to avoid being contacted unnecessarily."  [45] “It [ePRO] said that I’ve been having problems with my wound, and it said that I ought to see somebody within 48 hours. Well as I had an appointment…this morning at [the hospital] I didn’t see any need to call anybody.”  [50] "Even though I had pain, I didn’t think it was serious enough. I saw no reason to send an alert because I knew all along that this would take time." |
| **Instant feedback on symptoms after finishing the assessment** | [53] “It would be better to have my result instantly. Now just the doctors in public hospitals know our health condition, but if we know too, we can double check with the other doctors in private hospitals. This report should be seen as my own property.” |
| **Better push symptom management messages** | [51] “Yeah definitely. I can’t remember what they say exactly but from memory it was just the first time I read it either. Yeah. And then I noticed after a few days was the same thing so I just I was okay with that. And when it changed color I read it again. Come on. OK it’s like something different again. I think that, like I said before the more personable thing that’s definitely something to look at.”  [55] "The information section, I would rather have that more generalized and categorized. It is easier for the person to use.”  [55] "It would help if you could customize to the individual...the specific therapy they are receiving...There is one major thing, that is the food. One of the problems in my experience, I went through two different regimens of chemotherapy, different elements of the regimens have different food requirements, some for example do not allow caffeine or alcohol or meat products or spices. None of that is here, that would be helpful if me and my wife are about to contemplate dinner and it tells us can I eat such and such, those answers would be helpful in terms of my chemotherapy. This app tells us about chemotherapy in general, whereas different patients have different regimens of chemotherapy..." |
| **Better interface settings** | [46] “I don’t know, something that makes me want to not be so, I guess, clinical. Um, make it a little more happy.”  [53] “The screen seemed to be too monotonous. If there are some cartoons or color around, it will not be so dull. You can make a decorative border. Maybe after finishing one part, a cartoon will jump out. It will be cuter. This can also act as a break time for you to take a rest before going to the next part. Now it just looks like an examination.”  [55] "...add a search button. Having a search button just kills so much time. You can access the entire database in like 2 seconds. Saves a lot of time...I wish it had a search button."  [55] "It is always nice for someone in my age if got a bigger text...Of course, if you could make it bigger, would be great." |
